# Identification of dehydrogenase, hydratase, and aldolase responsible for the propionyl residue removal in degradation of cholic acid C-17 side chain in *Comamonas testosteroni* TA441

**DOI:** 10.1128/spectrum.00308-25

**Published:** 2025-09-15

**Authors:** Masae Horinouchi

**Affiliations:** 1 Surface and Interface Science Laboratory, RIKEN 13593, Wako, Saitama, Japan; University of Huddersfield, Huddersfield, United Kingdom

**Keywords:** *Comamonas testosteroni*, bile acid, cholic acid, testosterone, cholesterol, steroid, brain-gut-microbiome axis, brain-gut axis, *Mycobacterium tuberculosis*, AlphaFold

## Abstract

Bacterial steroid degradation is gaining attention for its diverse roles, such as *Mycobacterium tuberculosis*’s reliance on the degradation of the C17 side chain of cholesterol for survival in host environments. ORF40–44 of *Comamonas testosteroni* TA441, previously characterized for its A-, B-, C-, and D-ring cleavage pathways, were hypothesized to correspond to the *igr* operon of *M. tuberculosis*, encoding the dehydrogenase ChsE1E2, hydratase ChsH1H2_MaoC_, and aldolase Ltp2 ChsH2_DUF35_, responsible for propionyl residue removal in the degradation of the cholic acid C17 side chain. However, low amino acid identity between the corresponding enzymes precluded functional assignment without experimental evidence. In this study, we generated gene-disrupted mutants of ORF40–44 and demonstrated that ORF41/ORF44, ORF40/ORF42, and ORF43 encode the dehydrogenase, hydratase, and aldolase, respectively. ORF40 encodes a bifunctional protein comprising MaoC and DUF35 domains. The MaoC domain of ORF40 and the ORF42-encoded protein form the hydratase, while the DUF35 domain is essential for aldolase activity. A mutant expressing ORF40_MaoC_ and ORF40_DUF35_ separately exhibited both hydratase and aldolase activities, suggesting these activities do not require a strictly formed complex of hydratase and aldolase. However, efficient propionyl residue removal appears to depend on the proper formation of each enzymatic complex, including ChsE1E2, ChsH1H2_MaoC_, and Ltp2ChsH2_DUF35_. Although (ChsE1-ChsE2)_2_ does not form a stable complex with (ChsH1-ChsH2_MaoC_)_2_-(Ltp2-ChsH2_DUF35_)_2_, some degree of interaction was suggested. AlphaFold-predicted three-dimensional structures of the TA441 enzymes and their complexes revealed striking similarities to those of *M. tuberculosis*, despite low amino acid identities. These findings shed light on the structural and functional conservation of bacterial steroid-degrading enzymes.

**IMPORTANCE:**

Research on bacterial steroid degradation began over 50 years ago, primarily to produce substrates for steroid drugs. Recently, the role of steroid-degrading bacteria in human health has garnered increasing attention. *Comamonas testosteroni* TA441 is a prominent model organism for studying aerobic steroid degradation, with its overall pathways for A-, B-, C-, and D-ring cleavage already elucidated. In this study, we identified the mechanism for removing the propionyl residue in the degradation of the cholic acid C17 side chain, a crucial step in degrading steroids with a C17 side chain, such as cholic acid, cholesterol, and other biologically significant compounds in animals and plants. The functions and structures of the identified enzymes show remarkable similarity to those in *Mycobacterium tuberculosis*. These findings suggest that insights gained from TA441 could provide valuable clues for understanding *M. tuberculosis* steroid metabolism and the broader ecological and health-related implications of bacterial steroid degradation.

## INTRODUCTION

Steroid compounds perform various functions in both plants and animals (1), including humans, where they play critical roles as hormones, cholesterol, and bile acids (2–4). These compounds have garnered increasing attention due to their impact on human health, particularly in the context of pathogenic bacteria (5, 6). For example, a cholesterol import system is essential for the persistence of *Mycobacterium tuberculosis* H37Rv in the lungs of chronically infected animals (7), and cholesterol catabolism is crucial for pathogen maintenance in the host (8).

Cholic acid and deoxycholic acid, the primary components of human bile acids, are secreted into the bile and later reabsorbed in the ileum, where approximately 95% of bile acids are reabsorbed. The remaining portion reaches the colon, where it is converted into secondary bile acids by resident bacteria, influencing human health (9). Recent studies have revealed the presence of bacterial flora in the jejunum and ileum, suggesting bile acid transformation by bacteria in these regions (10). The bacterial composition in the ileum differs from that in the colon, with ileal microbiotas showing dynamic adaptation to fluctuating intraluminal ecological conditions. However, studies on ileal bacteria are limited due to sampling difficulties.

The ability of bacteria to aerobically degrade steroids has been known for over 70 years, with pioneering studies on *Rhodococcus equi* and *Comamonas testosteroni* conducted in the 1960s (11–14). These studies identified major intermediates in the A- and B-ring degradation pathways and revealed a similar mechanism in both bacteria.

**Fig 1:**
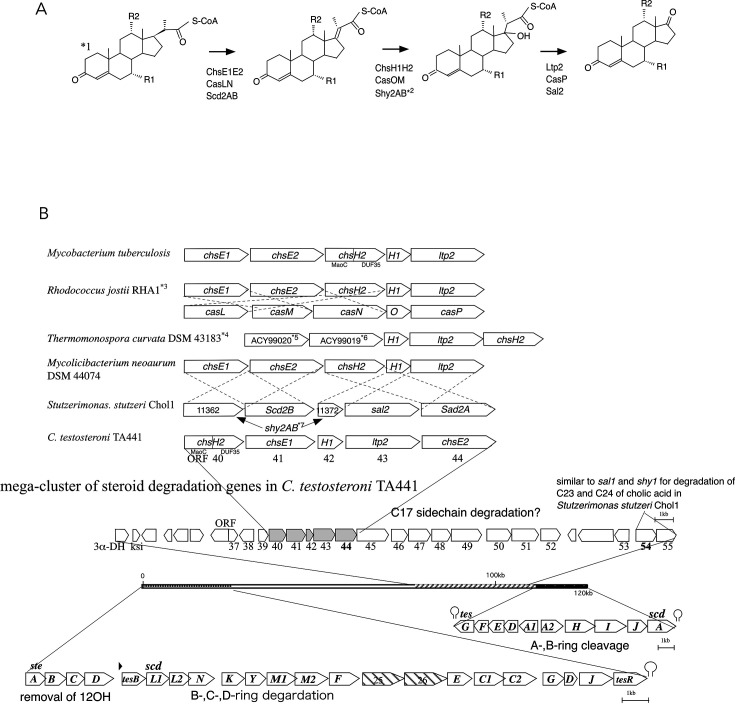
(**A**) The degradation process of the C20–22 propionyl residue in aerobic steroid degradation reported in Actinobacteria (*Mycobacterium tuberculosis* H37Rv [8, 30–35], *Rhodococcus jostii* RHA1 [28, 36], *Thermomonospora curvata* [35], and *Mycolicibacterium neoaurum* DSM 44074 [37], and the proteobacterium *Stutzerimonas stutzeri* Chol1 [partially identified] [38]). The initial reaction is catalyzed by a dehydrogenase (ChsE1E2 in *M. tuberculosis*, *T. curvata*, and *M. neoaurum*; CasLN in *R. jostii* RHA1; and Scd2AB in *S. stutzeri*), followed by the addition of a water molecule by a hydratase (ChsH1H2 in *M. tuberculosis*, *T. curvata*, and *M. neoaurum*; CasOM in *R. jostii* RHA1; and Shy2AB in *S. stutzeri*). Finally, the propionyl residue is removed by an aldolase (Ltp2 in *M. tuberculosis*, *T. curvata*, and *M. neoaurum*; CasP in *R. jostii* RHA1; and Sal2 in *S. stutzeri*). *1: the A-ring has a double bond at C4 in Actinobacteria except for *cas* genes and two double bonds at C1 and C4 in *P. stutzeri* and *R. jostii* RHA1’s *cas* genes. R1 and R2 represent H or OH groups. *2: speculation based on identity of aa (the substrate and the product have not been identified). (**B**) (Top) Comparison of *chsE1*, *E2*, *H1*, *H2*, and *ltp2* in *M. tuberculosis* with corresponding genes in other bacteria and ORF40–44 in *C. testosteroni* TA441. *3: *R. jostii* RHA1 has two sets of the genes; upper for cholesterol degradation and lower for cholic acid degradation. *4: *chsE1* and *E2* are not identified. *5: encoding a protein of unknown function DUF35. *6: encoding an acyl-CoA dehydrogenase. (Bottom) The 120 kb mega-cluster of steroid degradation genes in TA441. All the steroid degradation genes identified at present, except for 17β-dehydrogenase gene, are encoded within this mega-cluster. Two gene clusters for degradation of the sterane structure (steroidal ABCD rings), the gene cluster for A- and B-ring cleavage and for D- and C-ring degradation primarily via β-oxidation, are on both ends. The third cluster for C17 side-chain degradation, containing ORF40–44, is in the internal around 75 kb DNA region between these clusters. ORF54 and ORF55, which are similar to *sal1* and *shy1* in *Stutzerimonas stutzeri* Chol1 for removal of C23 and C24 in cholic acid degradation, are also in this region.

Our previous research elucidated the degradation pathways for the four steroidal rings (sterane; A-, B-, C-, and D-rings) in *C. testosteroni* TA441, which are encoded within a 120 kb mega-cluster of steroid degradation genes (Fig. 1B, below). The process begins with simultaneous B-ring cleavage and A-ring aromatization, followed by A-ring cleavage, all catalyzed by *tesA1A2BDEFHIJ* (15–22), and proceeds to degradation of the D- and C-rings primarily via β-oxidation, mediated by *scdAC1C2DEFGJKL1L2M1M2NY* (23–27). This pathway has established *C. testosteroni* TA441 as a prominent model organism for studying bacterial aerobic steroid degradation (an overview is provided in Fig. S1). Similar pathways are predicted to occur in other aerobic steroid-degrading bacteria, such as Rhodococci and Mycobacteria (28, 29).

**Fig 2:**
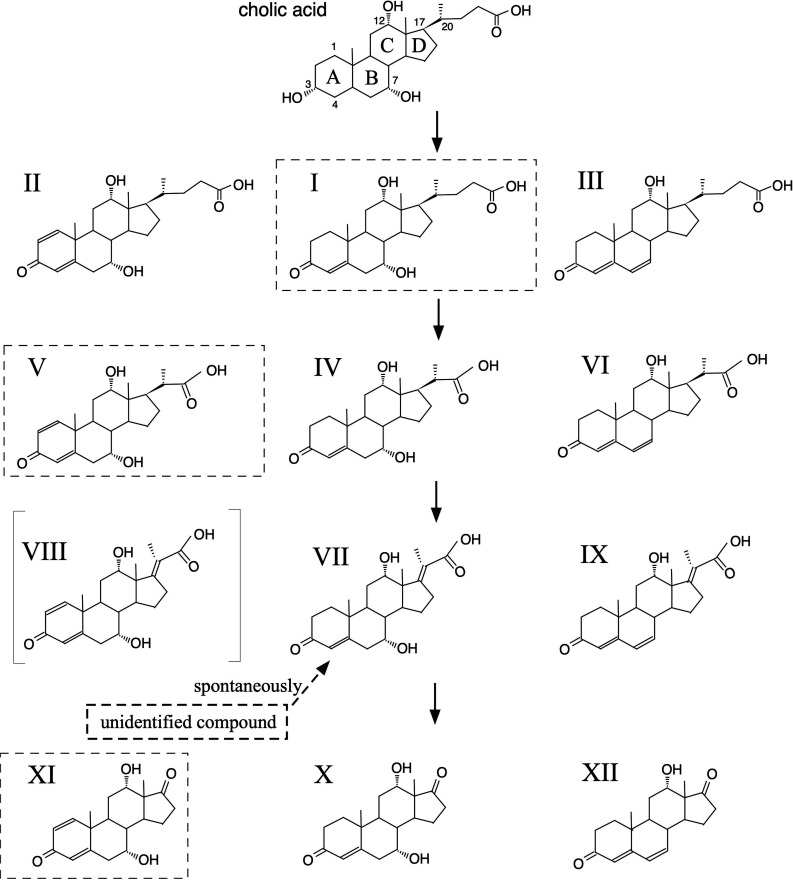
Compounds identified as metabolites of cholic acid during C17 side-chain degradation by *C. testosteroni* TA441 in a previous study. The compounds were isolated and identified using c and high-resolution mass spectrometry (23) (cf. Fig. S2). The actual metabolites in the degradation process are CoA esters. Compounds within broken squares were isolated in larger amounts than others with the same C17 side chain and are considered major metabolites. The compounds are cholic acid (3α,7α,12α-trihydroxy-5β-cholan-24-oic acid); 7α,12α-dihydroxy-3-oxo-5β−4-cholen-24-oic acid (**I**); 7α,12α-dihydroxy-3-oxo-5β-1,4-choladien-24-oic acid (**II**); 12α-hydroxy-3-oxo-5β−4,6-choladien-24-oic acid (**III**); 7α,12α-dihydroxy-3-oxo-4-pregnene-20-carboxylic acid (**IV**); 7α,12α-dihydroxy-3-oxo-1,4-pregnadine-20-carboxylic acid (**V**); 12α-hydroxy-3-oxo-4,6-pregnadiene-20-carboxylic acid (**VI**); 7α,12α-dihydroxy-3-oxo-4,17-pregnadiene-20-carboxylic acid (**VII**); 7α,12α-dihydroxy-3-oxo-1,4,17-pregnatrine-20-carboxylic acid (**VIII**); 12α-hydroxy-3-oxo-4,6,17-pregnatrine-20-carboxylic acid (**IX**); 7α,12α-dihydroxy-3,17-dioxo-4-androsten (**X**); 7α,12α-dihydroxy-3,17-dioxo-1,4-androstadien (**XI**); and 12α-dihydroxy-3,17-dioxo-4,6-androstadien (**XII**).

In addition to its ability to degrade steroidal rings, *C. testosteroni* TA441 also degrades various steroids, such as testosterone and cholic acid, with remarkable efficiency. While the pathways for the steroid rings have been well characterized, the mechanisms for degrading the cholic acid C17 side chain remain unclear. In previous studies, we identified intermediate compounds from the culture of a TA441 mutant cultivated with cholic acid (Fig. 2) (23). These intermediates suggest that degradation of the cholic acid C17 side chain occurs concurrently with A-ring dehydrogenation. However, some intermediates were too scarce to identify, and one spontaneously converted to 7α,12α-dihydroxy-5β-cholan-4,17-dien-24-oic acid (**VII**) during storage.

Cholic acid C17 side-chain degradation involves two main steps: removal of C23 and C24, followed by the removal of the C20–22 propionyl residue. The conversion of the C20–22 propionyl residue to a ketone is a fundamental process shared among bacteria capable of degrading steroids such as cholesterol, cholic acid, and ergosterol. This process has been described in actinobacteria such as *M. tuberculosis* H37Rv (8, 30–34, 39), *Rhodococcus jostii* RHA1 (28, 36), *Thermomonospora curvata* (35), and *Mycolicibacterium neoaurum* DSM 44074 (37), as well as the proteobacterium *Stutzerimonas* (formerly *Pseudomonas*) *stutzeri* Chol1 (partially identified) (38) (Fig. 1A and B; *chsE1E2* were not identified in the nearby DNA region in *T. curvata*). In *Pseudomonas stutzeri* Chol1, some genes are speculative and have not been identified. Therefore, complete C20–22 propionyl residue degradation in Proteobacteria is yet to be clarified.

The degradation of the C20–22 propionyl residue in *M. tuberculosis* H37Rv involves the introduction of a double bond at C17–20 by a dehydrogenase, followed by hydroxyl group addition at C17 by a hydratase. The isopropionyl residue is then cleaved by an aldolase, producing a ketone at C17 (Fig. 1A). The hydratase in *M. tuberculosis* is a heterotetramer of ChsH1 and MaoC-like protein from the N-terminal domain of ChsH2 (Fig. 1B) (33). The aldolase is a heterotetrameric complex of Ltp2 and a DUF35 protein derived from the C-terminal domain of ChsH2 (35).

Based on intermediate compounds isolated from *C. testosteroni* TA441 and observations of a mutant with a transposon in ORF44 accumulating substrates of the dehydrogenase (23), we hypothesized that TA441 utilizes a similar pathway. However, the low amino acid identity (30%–45%) between TA441’s proteins encoded by ORFs near ORF44 and the corresponding *M. tuberculosis* proteins prevented functional assignment without experimental evidence.

Steroid degradation by actinobacteria, particularly *Mycobacterium* spp., has been extensively studied due to its critical role in cholesterol metabolism and its impact on the survival of pathogenic bacteria within host cells. In contrast to *Mycobacterium*, whose relevance to cholesterol degradation and the infection is clear, steroid degradation by Proteobacteria remains less explored, despite its potential significance in environmental, biogeochemical, and biomedical contexts. *Comamonas testosteroni* and related Proteobacteria—such as *Pseudomonas* sp. (40), *Steroidobacter denitrificans* (41), and *Caenibius tardaugens* (42)—are known for their ability to degrade aromatic compounds, steroids, and other environmental substances. These bacteria are widely distributed in soil and aquatic environments and contribute to the degradation of such compounds in nature (43). *C. testosteroni* is recognized as a representative aerobic steroid degrader and a model organism for aerobic testosterone degradation. One study demonstrated its potential role in androgen biodegradation in aerobic sewage, showing a remarkable increase in 16S rRNA and catabolic genes of *C. testosteroni* following testosterone treatment (44). To the best of our knowledge, steroid degradation genes have been reported in many *C. testosteroni* strains. However, among at least 27 *Comamonas* spp., only *Comamonas thiooxydans* (45) and *Comamonas resistens* (46, 47) have been found to possess steroid degradation genes similar to those of *C. testosteroni*.

Given their ability to degrade steroids such as cholic acid, cholesterol, androgen, and ergosterol, steroid-degrading actinomyces and Proteobacteria may play important roles in environmental steroid removal, carbon cycling, and host-microbe interactions. Elucidating the mechanisms by which these bacteria process steroidal structures could enhance our understanding of microbial contributions to biogeochemical processes. The ability of Proteobacteria to degrade bile acids suggests that they may impact gut microbiome dynamics and contribute to metabolic regulation in the host. *C. testosteroni* is known as the most common pathogen of the genus, and in a review of 33 reported cases, the most common sites of infection were the bloodstream (13 cases) and the peritoneal cavity (10 cases) (48), which also suggests their presence in the gastrointestinal tract. However, the specific effects of proteobacterial steroid degradation on host physiology remain an open question, requiring further investigation.

Despite these potential roles, the molecular basis of aerobic steroid catabolism in Proteobacteria remained poorly understood until we identified a 120 kb steroid degradation mega-cluster in *C. testosteroni* TA441 (Fig. 1B [21, 22, 49]). Except for the 17β-dehydrogenase gene, all known steroid degradation genes in TA441 are located within this cluster. One end encodes genes for A- and B-ring cleavage, while the other end encodes genes for C- and D-ring degradation, primarily via β-oxidation. Between these regions lie the 3α-dehydrogenase and Δ4(5)-isomerase (ksi) genes (21), along with putative C17 side-chain degradation genes—including chs-like ORF40–44—occupying approximately a 75 kb region downstream of *tesG* (ORF55). ORF54 and ORF55 are homologous to *sal1* and *shy1* of *S. stutzeri* Chol1, which are involved in the removal of C23 and C24 during cholic acid catabolism (38). Transposon insertions in ORF44 or ORF54 abolish growth on cholic acid but not on testosterone, supporting their involvement in C17 side-chain degradation in TA441 (M. Horinouchi, unpublished data) (17). Notably, the downstream region of ORF44 is markedly different from that of Chol1 (40).

This study aims to bridge this knowledge gap by elucidating the function of key enzymes involved in C17 side-chain degradation in *C. testosteroni* TA441 and providing structural insights into their catalytic mechanisms. By comparing these enzymes with those of *M. tuberculosis*, this research seeks to reveal conserved and divergent features of bacterial steroid metabolism, offering new perspectives on its ecological, biogeochemical, and biomedical significance.

## RESULTS

### Analysis of intermediate compounds accumulated by gene-disrupted mutants

*Comamonas testosteroni* TA441 degrades cholic acid, a major component of bile acids. In our previous study, we elucidated the steroidal ABCD ring degradation process in TA441 (49, 50), which occurs after the removal of the C17 side chain. However, the mechanism for C17 side-chain degradation in TA441 has not yet been identified. In our earlier studies analyzing intermediate compounds of cholic acid in cultures of a TA441 mutant (*scdD*-disrupted mutant), compounds shown in Fig. 2 were purified and identified using nuclear magnetic resonance and mass spectrometry (23). Some of these compounds were unstable and converted to other derivatives. For example, one intermediate converted to 7α,12α-dihydroxy-5β-cholan-4,17-dien-24-oic acid (**VII**) under acidic conditions, suggesting it was 7α,12α,17-trihydroxy-5β-cholan-4-en-24-oic acid (Fig. 1A and 2). A TA441 transposon mutant with an insertion in ORF44 accumulated 7α,12α-dihydroxy-5β-cholan-4-en-24-oic acid (**V**) when cultured with cholic acid (23). To further investigate, we performed homology searches and found that the predicted amino acid sequences of ORFs 41, 44, 40, 42, and 43 shared 35%–63% identity with the dehydrogenase α- and β-subunit ChsE1E2, the hydratase α- and β-subunit ChsH1H2, and the retro-aldolase Ltp2 of *M. tuberculosis* H37Rv, respectively (Table 1). The identities between the corresponding enzymes in other bacterial genera—*Rhodococcus jostii*, *Thermomonospora curvata*, *Mycolicibacterium neoaurum*, and *Stutzerimonas stutzeri*—are summarized in Table 2. Therefore, to investigate the role of these genes, we constructed gene-disrupted mutants targeting ORF40–44 and analyzed cultures incubated with cholic acid using high-performance liquid chromatography with a three-dimensional (3D) UV detection system (Fig. S2-1). When this analysis was conducted, the mass spectrometry system was not available. We compared the retention time and pattern of UV absorption with those isolated from the culture of *scdD*-disrupted mutant (23), indicating that the major compounds in the cultures of the gene-disrupted mutants targeting ORF40–44 were intermediates in the propionyl residue degradation pathway.

**TABLE 1:** Strains^*a*^

Strain	Characteristics	Source or reference
TA441	Wild type	(51, 52)
ORF40(ChsH2) _MaoC_^−^	ORF40(*chsH2*)::Km^r^ mutant (inserted in MaoC domain) of TA441	This work
ORF40(ChsH2)_DUF35_^−^	ORF40(*chsH2*)::Km^r^ mutant (inserted in DUF35 domain) of TA441	This work
ORF41(ChsE1)^−^	ORF41(*chsE1*)^-^::Km^r^ mutant of TA441	This work
ORF42(ChsH2)^−^	ORF42(*chsH2*)::Km^r^ mutant of TA441	This work
ORF43(Ltp2)^−^	ORF43(*ltp2*)::Km^r^ mutant of TA441	This work
ORF44(ChsE2)^−^	ORF44(*chsE2*)::Km^r^ mutant (inserted in *Eco*RV site) of TA441	This work
ChsE1^–^H1^–^H2^–^Ltp2^–^	ORF40, 41, 42, 43(*chsE1,H1,H2,ltp2*): :Km^r^ mutant of TA441	This work

^*a*^ Km^r^, kanamycin resistance.

**TABLE 2:** Identities to corresponding enzymes in *C. testosteroni*

Bacteria	ChsE1	ChsE2	ChsH1	ChsH2	Ltp2
*Mycobacterium tuberculosis*	31%	44–49%	34–38%	43–44%	61–64%
*Rhodococcus jostii* ^*-*^	31–35%	46–49%	32–38%	49–50%	61–62%
*Thermomonospora curvata* ^*-*^	33–36%	45–47%	39%	55%	64%
*Mycolicibacterium neoaurum* ^*-*^	33%	46–47%	34–36%	43–44%	61–62%
*Stutzerimonas stutzeri*	49–53%	69–73%	60–66%	61–63%	80–83%

ChsE1E2, ChsH1H2, and Ltp2 in *M. tuberculosis*, encoded by the *igr* operon (*chsE1* to *ltp2*, Fig. 1B), are well studied for their roles in the propionyl residue degradation pathway (30–34). ChsE1 and ChsE2 form α_2_β_2_ heterotetramers with two active sites regulated by cholesterol (31, 32). ChsH2 comprises MaoC-like dehydratase and DUF35 domains (Fig. 1B), and ChsH2_MaoC_ and ChsH1 form a hydratase complex, while ChsH2_DUF35_ and Ltp2 form an aldolase complex, both as α_2_β_2_ heterotetramers (33, 35). In TA441, the MaoC-like domain and DUF35 domain were found in ORF40. Consequently, the MaoC-like and DUF35 domains of ORF40 were analyzed separately.

**Fig 3:**
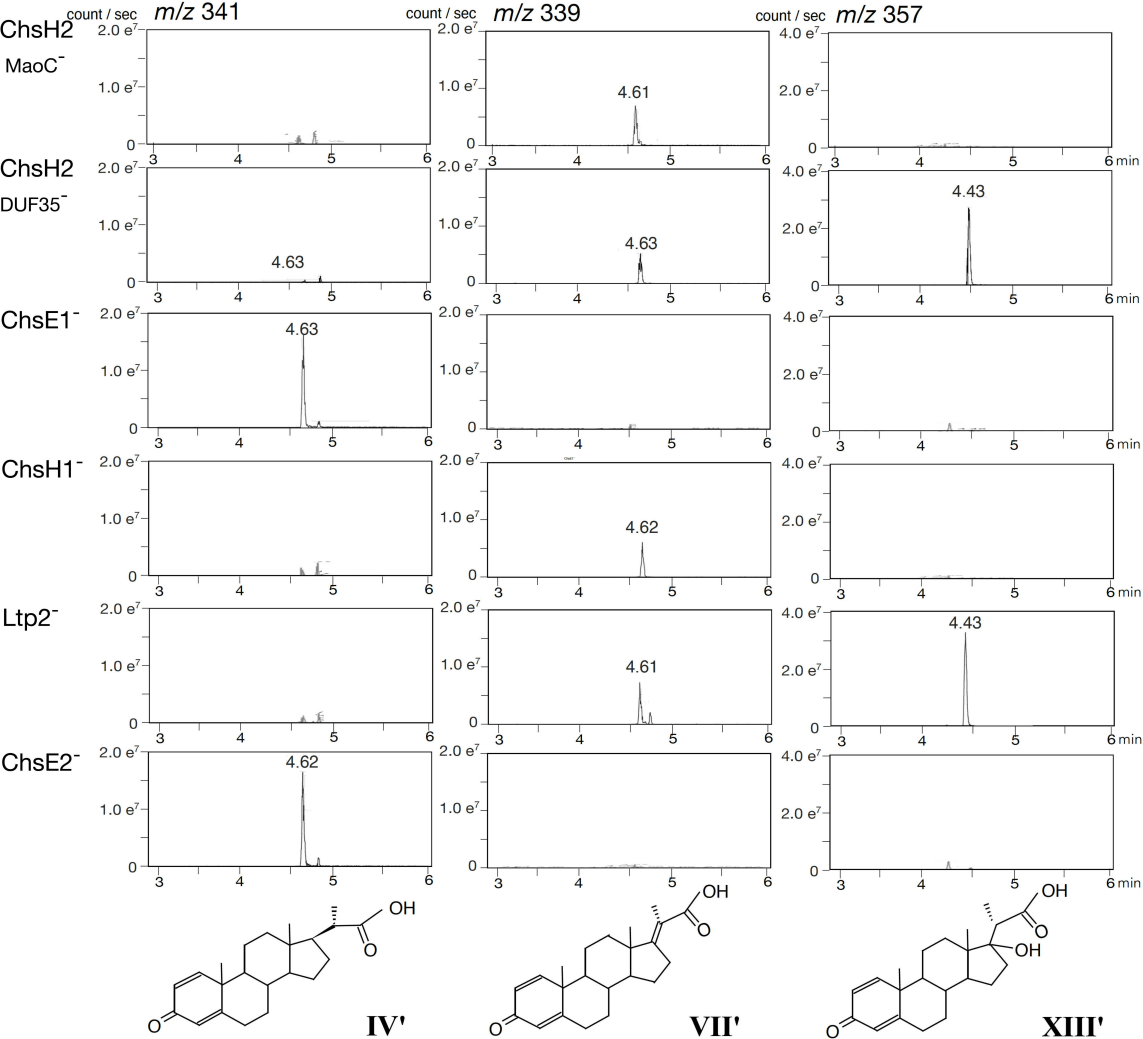
Analysis of TA441 mutants disrupted in ChsH2 to ChsE2, incubated with 0.05% lithocholic acid for 7 days. Mutants were constructed by inserting a kanamycin resistance gene. For ChsH2, separate mutants targeting the MaoC domain (ChsH2_MaoC_^–^) and the DUF35 domain (ChsH2_DUF35_^–^) were created. Peaks in mass spectra represent *m*/*z* 341 at retention time (RT) = 4.6 min (3-oxo-4-pregnene-20-carboxylic acid, **IV′**); *m/z* 339 at RT = 4.6 min (3-oxo-4,17-pregnadiene-20-carboxylic acid, **VII′**); and *m*/*z* 357 at RT = 4.4 min (17-hydroxy-3-oxo-1,4-pregnatriene-20-carboxylic acid, **XIII′**). The vertical axis indicates intensity (counts/s), and the horizontal axis indicates retention time (min).

To determine whether the proteins encoded by ORF40–44 function similarly to their counterparts in *M. tuberculosis*, we disrupted ORF40_MaoC_, ORF40_DUF35_, ORF41, ORF42, ORF43, and ORF44 using kanamycin resistance gene insertions (ORF40_MaoC_^−^, ORF40_DUF35_^−^, ORF41^−^, ORF42^−^, ORF43^−^, and ORF44^−^) (Table 3). Cultures of these mutants were analyzed using ultra-high-performance liquid chromatography/mass spectrometry (UPLC/MS) after incubation with cholic acid. However, the complexity of the resulting peaks, due to the three hydroxyl groups (C3, C7, and C12) of cholic acid producing multiple derivatives (see Fig. 2), necessitated the use of lithocholic acid (LCA) instead. LCA has only one hydroxyl group at C3 that is typically converted to a ketone group before the C17 side-chain degradation of cholic acid. The mutants were individually incubated with LCA, and the resulting cultures were analyzed using UPLC/MS (Fig. 3). In the cultures of ORF41^−^ and ORF44^−^, 3-oxo-1,4-pregnene-20-carboxylic acid (**IV′**) accumulated as the major intermediate compound. In contrast, 3-oxo-1,4,17-pregnene-20-carboxylic acid (**VII′**) was detected in the cultures of ORF40_MaoC_^−^, ORF40_DUF35_^−^, ORF42^−^, and ORF43^−^, with **VII′** being the predominant compound in the cultures of ORF40_MaoC_^−^ and ORF42^−^. Additionally, 17-hydroxy-3-oxo-1,4-pregnadiene-20-carboxylic acid (**XIII′**) accumulated in large amounts in the cultures of the ORF40_DUF35_^−^ and ORF43^−^ mutants. Details of the identification process for these intermediate compounds are provided in Fig. S2-1 to S2-4. **VII′** was detected in the cultures of ORF40_DUF35_^−^ and ORF43^−^, likely because the excessive accumulation of **XIII′** hindered the conversion of **VII′** to **XIII′**. The detected amount of **VII′** was relatively small compared to **IV′** and **XIII′**, consistent with our previous study in which **VII** was identified as an unstable intermediate (23). During isolation, **VII** rapidly degraded, making its identification challenging. This instability could partially explain the smaller amounts of **VII′** observed in this study. In TA441, the major intermediate compounds were derivatives with two double bonds at C1 and C4 in the A-ring. This contrasts with *M. tuberculosis*, where intermediates with a single double bond at C4 are predominant.

**TABLE 3:** Features of ORFs 40–44

ORF	Putative function of the encoded enzyme / putative substrate	Corresponding gene in *M. tuberculosis*	a.a. identity (%)
ORF40	Hydratase α subunit / 3-oxo-4-pregnene-20-carboxyl-CoA (3-oxo-23,24-bisnorchol-4,17(20)-dien-22-oyl-CoA)	*chsH2*	35
ORF41	Dehydrogenase α subunit / 3-oxo-4,17-pregnadiene-20-carboxyl-CoA (3-oxo-23,24-bisnorchol-4-en-22-oyl-CoA)	*chsE1*	31
ORF42	Hydratase β subunit	*chsH1*	44
ORF43	Aldolase / 17-hydroxy-3-oxo-4-pregnene-20-carboxyl-CoA	*trp2*	63
ORF44	Dehydrogenase β subunit	*chsE2*	44

These findings support the hypothesis derived from homology searches that ORF41 and ORF44 encode 3-oxo-4-pregnene-20-carboxyl-CoA dehydrogenase (ChsE1-E2 in *M. tuberculosis*); ORF42 and ORF40_MaoC_ encode 3-oxo-4,17-pregnadiene-20-carboxyl-CoA hydratase (ChsH1-H2_MaoC_); and ORF43 and ORF40_DUF35_ encode an aldolase responsible for removing the isopropyl-CoA side chain (Ltp2-ChsH2_DUF35_); therefore, they were named ChsE1-E2, ChsH1-H2_MaoC_, and Ltp2-ChsH2_DUF35_, respectively. ChsH2_DUF35_ is indispensable for aldolase activity, while the hydratase encoded by ChsH1 and ChsH2_MaoC_ remains active even in the absence of the DUF35 domain of ChsH2. To better understand the specific roles and interactions of these subunits, we conducted complementation experiments to further explore their functions and interdependencies.

### Complementation experiments involving gene-disrupted mutants ChsE1^–^, ChsE2^–^, ChsH1^–^, ChsH2_MaoC_^–^, ChsH2_DUF35_^–^, and Ltp2^–^

**Fig 4:**
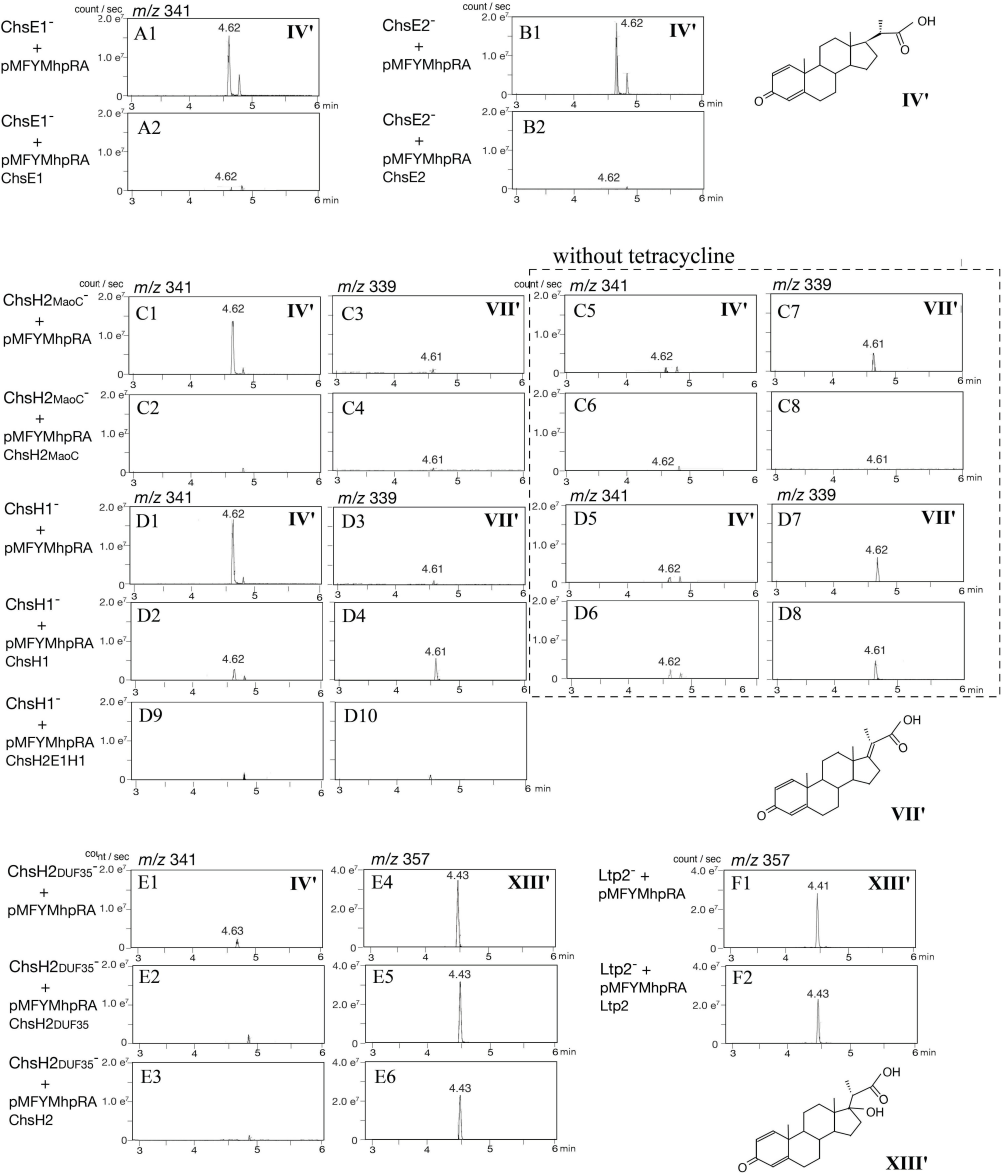
Complementation experiments with mutants disrupted in ChsH2 to ChsE2, incubated with 0.05% lithocholic acid for 7 days. Mass chromatograms of each mutant are presented, showing peaks at *m*/*z* 341 (**IV′**), *m*/*z* 339 (**VII′**), and *m*/*z* 357 (**XIII′**). Peaks were undetectable when the data were not presented. The chromatograms within broken squares represent cultures without tetracycline. Mutants are ChsE1^−^ carrying pMFYMhpRA (ChsE1^−^ with pMFYMhpRA) (A1) and pMFYMhpChsE1 (pMFYMhpRA-derivative carrying ChsE1) (A2), ChsE2^−^ carrying pMFYMhpRA (ChsE2^−^ with pMFYMhpRA) (B1) and pMFYMhpChsE2 (pMFYMhpRA-derivative carrying ChsE2) (B2), ChsH2_MaoC_^−^ carrying pMFYMhpRA (ChsH2_MaoC_^−^ with pMFYMhpRA) (C1, C3, C5, and C7), ChsH2_MaoC_^−^ carrying pMFYMhpChsH2_MaoC_ (pMFYMhpRA-derivative with ChsH2_MaoC_) (C2, C4, C6, and C8), ChcH1^−^ carrying pMFYMhpRA (ChcH1^−^ with pMFYMhpRA) (D1, D3, D5, and D7), ChcH1^−^ carrying pMFYMhp ChcH1 (pMFYMhpRA-derivative with ChcH1) (D2, D4, D6, and D8), ChcH1^−^ carrying pMFYMhpChsE1H1H2 (pMFYMhpRA-derivative with ChsE1H1H2) (D9 and D10), ChsH2_DUF35_^−^ carrying pMFYMhpRA (ChsH2 _DUF35_^−^ with pMFYMhpRA) (E1 and E4), ChsH2 _DUF35_^−^ carrying pMFYMhpChsH2 _DUF35_ (pMFYMhpRA-derivative with ChsH2_DUF35_) (E2 and E5), ChsH2 _DUF35_^−^ carrying pMFYMhpChsH2_DUF35_ (pMFYMhpRA-derivative with ChsH2) (E3 and E6), Ltp2 carrying pMFYMhpRA (Ltp2 ^−^ with pMFYMhpRA) (F1), and Ltp2 ^−^ carrying pMFYMhpLtp2 (pMFYMhpRA-derivative with Ltp2) (F2).

To investigate the roles of ChsE1E2H1H2 and Ltp2, complementation experiments were conducted using mutants carrying plasmids derived from the broad-host-range vector pMFYMhpRA (50), a derivative of pMFY42 (53). The mutants were constructed as follows: ChsH2_MaoC_^–^ carrying pMFYMhpRA (negative control), ChsH2_MaoC_^–^ carrying a plasmid encoding ChsH2_MaoC_ (pMFYMhpChsH2_MaoC_), ChsH2_DUF35_^–^ carrying pMFYMhpRA (negative control), ChsH2_DUF35_^–^ carrying pMFYMhp ChsH2_DUF35_, and similarly for ChsE1^–^, ChsH1^–^, Ltp2^–^, and ChsE2^–^ with their respective complementing plasmids (Table S2; Fig. 4). Peaks were undetectable when the data were not presented in Fig. 4.

In the cultures of ChsE1^–^ and ChsE2^–^ mutants carrying pMFYMhpRA (negative controls), **IV′** accumulated as the major intermediate (Fig. 4A1 and B1), and **IV′** was degraded in the complemented mutants carrying pMFYMhpChsE1 and pMFYMhpChsE2 (Fig. 4A2 and B2). Similarly, **XIII′** accumulated in ChsH2_DUF35_^–^ and Ltp2^–^ mutants carrying pMFYMhpRA (Fig. 4E4 and F1). The amount of **XIII′** was reduced in cultures of ChsH2_DUF35_^–^ carrying pMFYMhp ChsH2_DUF35_ and Ltp2 ^–^ carrying pMFYMhpLtp2, although the reduction was modest (Fig. 4E5 and F2). To further investigate, we constructed a new mutant, ChsH2_DUF35_^–^ carrying pMFYMhpChsH2, and observed more pronounced reduction in **XIII′** compared to pMFYMhpChsH2_DUF35_ (Fig. 4E6). To our surprise, **IV′** unexpectedly accumulated in the cultures of the ChsH2_MaoC_^−^ carrying pMFYMhpRA and the ChsH1^−^ carrying pMFYMhpRA (Fig. 4C1 and D1), even though **VII′** had been observed as the major accumulated compound in previous experiments with these mutants. This discrepancy was thought to be attributed to the presence of tetracycline, which was added to maintain the plasmids in this experiment. To confirm this, the same experiments were repeated without tetracycline (Fig. 4C5 to C8 and D5 to D8). Under these conditions, **VII′** was the major compound detected in the cultures of ChsH2_MaoC_^−^ carrying pMFYMhpRA and the ChsH1^−^ carrying pMFYMhpRA (Fig. 4C7 and D7). Tetracycline functions by inhibiting protein synthesis in bacteria through its interaction with the bacterial 30S ribosomal subunit, while the tetracycline-resistance gene encodes a pump that expels tetracycline from the cell. If tetracycline had inhibited the expression of the dehydrogenase ChsE1 and ChsE2, **IV′** should have accumulated in the ChsH2_DUF35_^−^ and Ltp2^−^ carrying pMFYMhpRA. Therefore, the possibility of direct inhibition of the dehydrogenase by tetracycline was ruled out. The mechanism underlying the accumulation of **IV′** in the cultures of ChsH2_MaoC_^−^ and ChsH1^−^ carrying pMFYMhpRA remains unclear. However, it is possible that interactions between the dehydrogenase ChsE1 and ChsE2, and the hydratase ChsH2_MaoC_ and ChsH1, influence this outcome. In the culture of ChsH2_MaoC_^−^ complemented with pMFYMhp ChsH2_MaoC_, with tetracycline, as well as without tetracycline, the levels of both **VII′** and **IV′** were reduced to nearly undetectable levels (Fig. 4C2, C4 and C8). Similarly, in the cultures of the ChsH1^−^ carrying pMFYMhpRA with tetracycline, **IV′** levels were barely detectable after complementation with pMFYMhpChsH1 (Fig. 4D2). However, the reduction in **VII′** levels was minor in the cultures of ChsH1^−^ with pMFYMhpChsH1 (Fig. 4D4 and D8). In the culture of the ChsH1^−^ with a plasmid encoding ChsH2 to ChsH1 (pMFYMhpChsE1H1H2), both **IV′** and **VII′** were nearly undetectable (Fig. 4D9 and D10).

While the precise reasons for these observations in ChsH2_MaoC_^−^ and ChsH1^-^ with tetracycline remain unclear, the complementation experiments support the hypothesis that the dehydrogenase ChsE1 and ChsE2 convert **IV′**-CoA to **VII′**-CoA. This is followed by the addition of a water molecule at C17, catalyzed by the hydratase ChsH1 and ChsH2_MaoC_, and the removal of the C17 residue as a ketone group, mediated by the aldolase Ltp2 and ChsH2_DUF35_.

### Complementation experiments with ChsE1H1H2Ltp2-disrupted mutant (ChsE1^−^H1^−^H2^−^Ltp2^−^)

**Fig 5:**
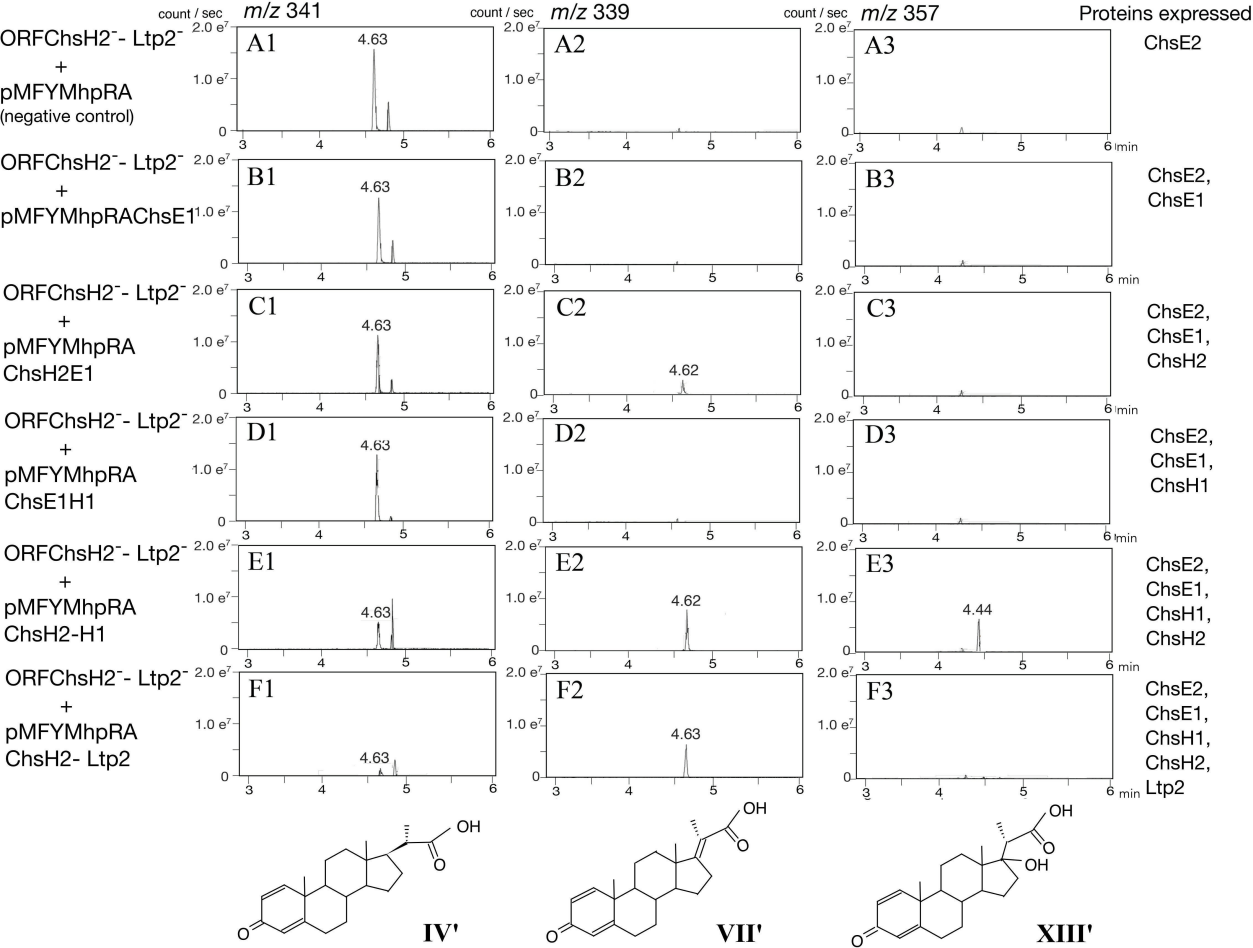
Complementation experiments with ChsE1H1H2Ltp2 disrupted mutants (ChsE1^–^H1^–^H2^–^Ltp2^–^) incubated with 0.05% lithocholic acid for 1 day, followed by the addition of 3-(3-hydroxyphenyl)propionic acid for gene induction and further incubation for 6 days. Mass spectra display peaks at *m*/*z* 341 (**IV′**), *m*/*z* 339 (**VII′**), and *m*/*z* 357(**XIII′**). ORFs expressed in each mutant are indicated on the right. Mutants are ChsE1^–^H1^–^H2^–^Ltp2^–^ carrying pMFYMhpRA (**A**), carrying pMFYMhp ChsE1 (**B**), carrying pMFYMhpChsH2E1 (pMFYMhpRA-derivative with ChsH2 and E1) (**C**), carrying pMFYMhpChsE1H1 (pMFYMhpRA-derivative with ChsE1 and H1) (**D**), carrying pMFYMhpChsE1H1H2 (pMFYMhpRA-derivative with ChsE1, H2, and H1) (**E**), and carrying pMFYMhpChsE1H1H2Ltp2 (pMFYMhpRA-derivative with ChsH2 to Ltp2) (**F**).

For further analysis, we attempted to construct a mutant with a complete disruption of *chsH2–chsE2* (*chsE1H1H2ltp2chsE2*). Despite multiple attempts using various plasmids designed for homologous recombination, all efforts to disrupt the entire *chsH2–chsE2* region were unsuccessful. Consequently, we constructed a *chsE1H1H2ltp2* disrupted mutant (ChsE1^–^H1^–^H2^–^Ltp2^–^) as an alternative. This mutant serves as a suitable replacement for ChsH2– ChsE2-disrupted mutants to investigate whether all three enzymes are indispensable for the removal of the propionyl residue, as the absence of either ChsE1 or ChsE2 abolishes dehydrogenase activity (Fig. 3 and 4). Then we respectively introduced pMFYMhpRAChsE1, a pMFYMhpRA-based plasmid encoding ChsH2 and ChsE1 (pMFYMhpChsE1H2), a pMFYMhpRA-based plasmid encoding ChsE1 and ChsH1 (pMFYMhpChsE1H1), pMFYMhpChsE1H1H2, a pMFYMhpRA-based plasmid encoding ChsH2 to Ltp2 (pMFYMhpChsE1H1H2Ltp2), and pMFYMhpRA (negative control) into the ChsE1^–^H1^–^H2^–^Ltp2^–^ (Table 1; Tables S1 and S2). The resulting mutants were incubated with LCA in the presence of tetracycline (Fig. 5). As observed in previous experiments, mutants lacking either ChsH2 or ChsH1 primarily accumulated **IV′**, except for ChsE1^–^H1^–^H2^–^Ltp2^–^ carrying pMFYMhpChsE1H2. This mutant, expressing ChsH2, ChsE1, and ChsE2, accumulated a small amount of **VII′**, indicating partial dehydrogenase activity even in the presence of tetracycline. Independent colonies of this mutant consistently produced similar results, confirming its partial activity in converting **IV′** to subsequent intermediates.

In the culture of ChsE1^–^H1^–^H2^–^Ltp2^–^ carrying pMFYMhp ChsE1H1H2, which expresses ChsE1E2H1H2, **IV′** was converted to **VII′** and **XIII′** (Fig. 5F), and cultures of ChsE1^–^H1^–^H2^–^Ltp2^–^ carrying pMFYMhpChsE1H1H2Ltp2, expressing all ChsE1E2H1H2Ltp2 proteins, showed nearly undetectable levels of **IV′** and **XIII′**, though **VII′** remained detectable (Fig. 5H). This result is consistent with previous findings using ChsH1^–^ carrying pMFYMhpChsH1 (Fig. 4D4 and D8), where **VII′** conversion was incomplete. ChsH2_DUF35_ was found to be indispensable for aldolase activity, whereas the ChsH1–ChsH2_MaoC_ hydratase retained activity even in the absence of the DUF35 domain of ChsH2. Despite the relatively low amino acid identity between the TA441 and *M. tuberculosis* enzymes (30%–45%, except for Ltp2), their functional and structural similarities are remarkable.

### AlphaFold prediction of the 3D structures of ChsE1E2H1H2Ltp2 in TA441 and the comparison with corresponding enzymes in *M. tuberculosis*

**Fig 6:**
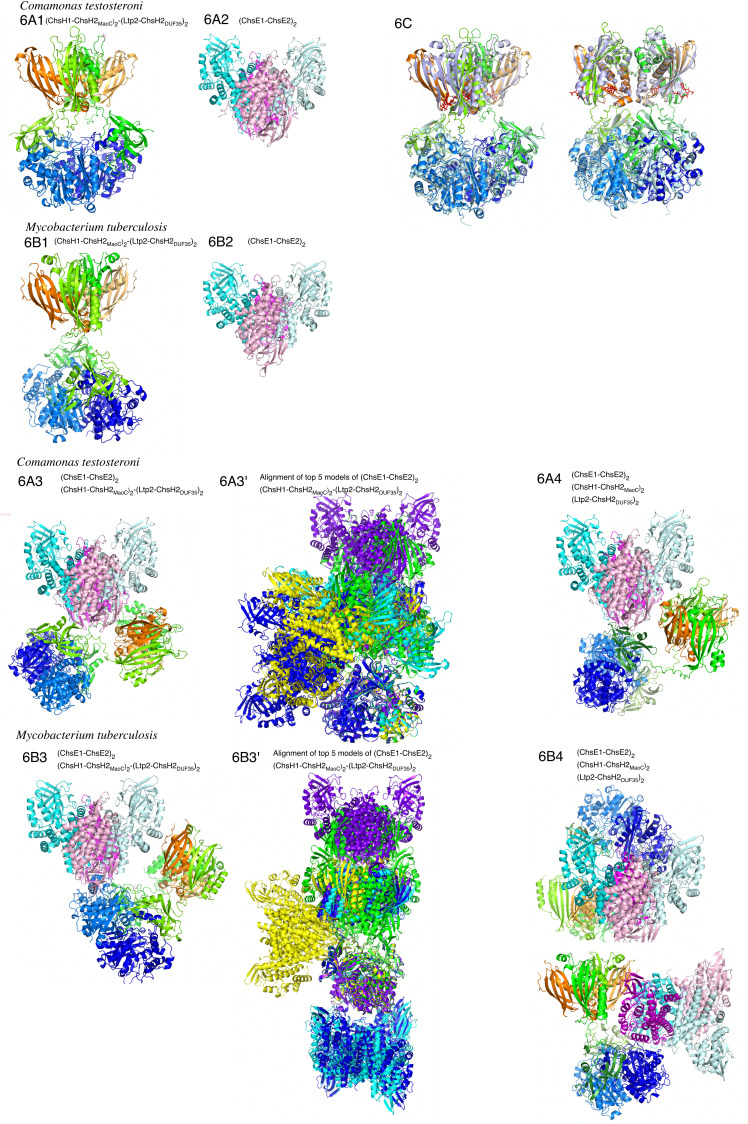
AlphaFold-predicted structures of ChsE1E2H1H2Ltp2 complex in *C. testosteroni* TA441 (A) and corresponding enzymes in *M. tuberculosis* (B). Complexes modeled include (ChsH1-ChsH2_MaoC_)_2_, (Ltp2-ChsH2DUF_35_)_2_, and (ChsE1-ChsE2)_2_, as well as their combined interactions. Predicted structures of proteins in other mutants are shown in Fig. S4. Expected positional errors are shown in Fig. S5. Color scheme: cyan/light cyan: ChsE1, magenta/light magenta: ChsE2, orange/light orange: ChsH1, green/light green: ChsH2 (or ChsH2_MaoC_ when divided), moss green/light moss green: ChsH2_DUF_, and blue/light blue: Ltp2. (A1, B1) (ChsH1_2_-ChsH2_MaoC_)_2_-(Ltp2-ChsH2_DUF35_)_2_; (A2, B2) (ChsE1-ChsE2)_2_; (A3, B3) (ChsE1-ChsE2)_2_ with (ChsH1_2_-ChsH2_MaoC_)_2_-(Ltp2-ChsH2_DUF35_)_2_; (A3′, B3′) alignment of top five models of (ChsE1-ChsE2)_2_ with (ChsH1_2_-ChsH2_MaoC_)_2_-(Ltp2-ChsH2_DUF35_)_2_ (color scheme: purple: the first model, dark blue: the second model, light blue: the third model, green: the fourth model, yellow: the fifth model); (A4, B4) (ChsE1-ChsE2)_2_ with (ChsH1_2_-ChsH2_MaoC_)_2_-(Ltp2-ChsH2_DUF35_)_2_, ChsH2_MaoC_ and ChsH2_DUF35_ are separately expressed. (B4) The same model with a different angle. (C) (ChsH1-ChsH2-Ltp2) _2_ complex of TA441 aligned with (ChsH1-ChsH2)_2_ complex of *M. tuberculosis* with 3-oxo-4-pregnene-20-carboxyl-CoA (**IV′**-CoA) (PDB 4wnb) and (Ltp2-ChsH2_DUF35_)_2_ complex of *T. curvata* (PDB 6ok1), the same model with a different angle. Complexes 4wnb and 6ok1 are in pale blue and **IV′**-CoA in red.

The 3D structures of the (ChsE1-ChsE2)_2_ dehydrogenase, (ChsH1-ChsH2_MaoC_)_2_ hydratase, and (Ltp2-ChsH2_DUF35_)_2_ aldolase of *M. tuberculosis* have been elucidated through crystal structure analyses (32, 34, 35). Using AlphaFold (54), we predicted the 3D structure of the (ChsH1-ChsH2_MaoC_)_2_ and (Ltp2-ChsH2_DUF35_)_2_ complexes in *C. testosteroni* TA441, as shown in Fig. 6A1 (with pLDDT score and alignment of top five models illustrated in Fig. S3). Expected position errors are illustrated in Fig. S5. This model closely resembles the docking model of the (ChsH1-ChsH2_MaoC_)_2_ complex of *M. tuberculosis* and the (Ltp2-ChsH2_DUF35_)_2_ complex of *Thermomonospora curvata* (35), despite slight differences in spatial arrangement. For example, the spacing between the (ChsH1–ChsH2_MaoC_)_2_ and (Ltp2–ChsH2_DUF35_)_2_ complexes in TA441 is slightly greater than that observed in *M. tuberculosis* (cf. Fig. 6C). In this model, the (ChsH1–ChsH2_MaoC_)_2_ complex of *M. tuberculosis* and the (Ltp2–ChsH2_DUF35_)_2_ complex of *T. curvata* are individually aligned with the (ChsH1–ChsH2_MaoC_)_2_–(Ltp2–ChsH2_DUF35_)_2_ complex model of TA441. Comparison with AlphaFold models of the (ChsH1–ChsH2–Ltp2)_2_ complex in *M. tuberculosis* revealed that the (Ltp2–ChsH2_DUF35_)_2_ complex is rotated approximately 90° counterclockwise relative to the (ChsH1–ChsH2_MaoC_)₂ complex (Fig. 6A1 and B1). Although the pLDDT scores for the region connecting these two complexes are lower than those for other areas, all five AlphaFold-generated models of the TA441 and *M. tuberculosis* (ChsH1–ChsH2–Ltp2)_2_ complexes displayed the same angular relationship (Fig. S3). This angular difference is unlikely to impact catalytic activity, as experiments showed that separately expressed ChsH2_MaoC_ and ChsH2_DUF35_ retained hydratase and aldolase activities (predicted structure [Fig. 6A4] and experimental data [Fig. 4C4]). Another 3D model of the (ChsH1-ChsH2-Ltp2) complex of *M. tuberculosis* was presented based on small-angle X-ray scattering and single-particle electron microscopy data (34). In this model, ChsH1-ChsH2 and Ltp2 of *M. tuberculosis* form a protomer, and two protomers were docked with Rosetta local refinement to present a model where two protomers joined symmetrically with respect to a point at Ltp2. Notably, all five AlphaFold-generated models of the TA441 (ChsH1–ChsH2–Ltp2)_2_ complex aligned closely with the structure presented in Fig. 6A1 (Fig. S3B). Figure 6C shows this TA441 complex aligned with the (ChsH1–ChsH2)_2_ complex of *M. tuberculosis* bound to 3-oxo-4-pregnene-20-carboxyl-CoA (PDB: 4wnb) and the (Ltp2–ChsH2_DUF35_)_2_ complex of *T. curvata* (PDB: 6ok1). The structural alignment was strong, suggesting that the substrate of TA441’s (ChsH1–ChsH2)_2_ complex—3-oxo-4-pregnene-20-carboxyl-CoA (**IV′**-CoA)—likely binds at the same site as in the *M. tuberculosis* complex.

The AlphaFold model of the (ChsE1-ChsE2)_2_ dehydrogenase of TA441 (Fig. 6A2) was also found to be structurally similar to its counterpart in *M. tuberculosis* (Fig. 6B2) (32). Our experimental data obtained in this study suggested some interaction between the (ChsH1-ChsH2-Ltp2)_2_ and (ChsE1-ChsE2)_2_ complexes (Fig. 4C and D). Therefore, we performed AlphaFold modeling with all the subunits, ChsH1, ChsH2, Ltp2, ChsE1, and ChsE2 (representative model is shown in Fig. 6A3, alignment of top five models is shown in Fig. 6A3′). When all the subunits were analyzed together, AlphaFold modeling revealed that the (ChsE1-ChsE2)_2_ dehydrogenase complex is positioned so close to Ltp2 that it causes a distortion in the (ChsH1-ChsH2_MaoC_)_2_-(Ltp2-ChsH2_DUF35_)_2_ complex. These findings suggest that although the (ChsE1–ChsE2)_2_ complex does not form a stable association with the (ChsH1–ChsH2_MaoC_)_2_–(Ltp2–ChsH2_DUF35_)_2_ complex, some degree of interaction likely occurs. This interaction may destabilize the latter complex but could facilitate the efficient removal of the C17 propionyl residue. In contrast, the (ChsH1–ChsH2_MaoC_)_2_–(Ltp2–ChsH2_DUF35_)_2_ complex in *M. tuberculosis* appears to be more stable (a representative model is shown in Fig. 6B3; alignment of the top five models is shown in Fig. 6B3′). In four out of the five models, the structure and positioning of the complex were consistently maintained.

When ChsH2_MaoC_ and ChsH2_DUF35_ were expressed separately, the distortion of the (ChsH1–ChsH2_MaoC_)_2_–(Ltp2–ChsH2_DUF35_)_2_ complex became more pronounced (Fig. 6A4). Despite this structural disruption, both hydratase and aldolase activities were retained, as shown in Fig. 4C1 and C2. Additional AlphaFold models of the complex in gene-disrupted mutants, along with their enzymatic activities, are presented in Fig. S4. Although the predicted positional error is high, the modeled enzyme complex structures are consistent with their observed activities (Fig. S4 and S5). The reason for the observed tetracycline-induced reduction in ChsH1H2 activity remains unclear. However, given that tetracycline inhibits protein synthesis, we cannot rule out the possibility that it affects the expression levels of the relevant proteins, which may, in turn, influence their folding or assembly and thereby affect the positioning of the (ChsE1–ChsE2_2_ and (ChsH1–ChsH2_MaoC_)_2_–(Ltp2–ChsH2_DUF35_)_2_ complexes.

## DISCUSSION

In this study, we identified the functions of ChsE1E2H1H2Ltp2 in *C. testosteroni* TA441 by constructing gene-disrupted mutants and comparing them with the corresponding Chs enzymes in *M. tuberculosis. chsE1* and *chsE2* were shown to encode the dehydrogenase responsible for converting 3-oxo-1,4-pregnediene-20-carboxyl-CoA to 3-oxo-1,4,17-pregnatriene-20-carboxyl-CoA (ChsE1-ChsE2 in *M. tuberculosis*). *chsH1* and *chsH2_MaoC_* encode the hydratase that adds a water molecule at C17 (ChsH1-ChsH2_MaoC_), while *ltp2* and *chsH2_DUF35_* encode the aldolase that removes the isopropyl-CoA side chain at C17 (Ltp2-ChsH2_DUF35_).

The structural and functional similarities between TA441 and *M. tuberculosis* enzymes highlight the evolutionary conservation of the steroid degradation pathway. We previously identified the genes and pathways involved in the degradation of sterane in TA441 (Fig. 1B, bottom; Fig. S1) (49, 50). Further comparative analyses of AlphaFold structures from other steroid-degrading bacteria, combined with experimental validation, may uncover universal mechanisms governing steroid degradation and its ecological and biomedical significance.

Homology searches revealed that a large number of enzymes in Pseudomonadota (Proteobacteria), most of which have not been explicitly identified, show similarity to ChsH1H2E1E2Ltp2. The homology search results suggest that steroid degradation by Proteobacteria is widespread in the environment. The ability of bacteria to degrade steroid compounds, including bile acids, may play a role in mitigating environmental pollutants and contribute to waste treatment processes. Understanding the molecular mechanisms involved in these degradations could provide insights into novel bioremediation strategies, where bacteria are utilized to degrade harmful steroid-based contaminants in polluted environments. From a biomedical perspective, the ability of certain bacteria to degrade steroids suggests potential implications for host-microbe interactions, especially in the human gut microbiome. The degradation of bile acids, which are essential for lipid digestion and absorption, influences not only host metabolism but also microbial dynamics in the gut. Furthermore, microbial degradation of steroid hormones could impact various physiological processes, such as infection and immune response. Understanding these microbial steroid degradation pathways may lead to novel therapeutic approaches for diseases related to steroid metabolism.

## MATERIALS AND METHODS

### Culture conditions

Mutant strains of *Comamonas testosteroni* TA441 were cultured at 30°C in a medium composed of equal volumes of Luria-Bertani (LB) medium and C medium, a mineral medium optimized for TA441 (15). This mixed medium was used because it facilitates the accumulation of intermediate compounds in mutants more effectively than either C medium or LB medium alone (unpublished data). LCA was added as a filter-sterilized solution in dimethyl sulfoxide, with a final concentration of 0.05% (wt/vol). LCA, not lithocholate, was added to culture as C medium works as an effective buffer and keeps the pH around 7. Similarly, 3-(3-hydroxyphenyl)propionic acid (3HPP) was prepared as an acetonitrile solution and added to the medium at a final concentration of 0.1% (wt/vol). The culture is incubated for 7 days for liquid chromatography/mass spectrometry analysis when anything else was not mentioned. 3HPP is added a day after the start of incubation when anything else was not mentioned because it can prevent the growth of TA441 if added at the start of incubation.

### Construction of deletion mutants, plasmids, and mutants for complementation experiments

To construct the ChsH2_MaoC_– mutant (Table 3), an *Hpa*I site was introduced into ChsH2_MaoC_ on plasmid pHSG10-2-8-28 (Table S1) using primers ORF40_Km^r^ and ORF40_Km^r^RC (Table S2). The kanamycin resistance (Km^r^) gene was subsequently inserted into the *Hpa*I site, resulting in the construction of pUC19-based (55) plasmid pUC ORF40-Km^r^ (ChsH2::Km^r^). The plasmid was introduced into *C. testosteroni* TA441 cells via electroporation, and transformants were selected on LB plates containing kanamycin (400 µg/mL). The insertion of Km^r^ into ChsH2 was confirmed by PCR amplification using DNA extracted from the resultant transformants.

Deletion mutants for ChsE1 through Ltp2 were constructed using similar methods with appropriate plasmids and primers listed in Tables S1 and S2. For the construction of pUCORF44-Km^r^, the Km^r^ gene was inserted into the *Eco*RV site within ChsE2. To disrupt ChsH2 through Ltp2, plasmid pUCORF40-43-Km^r^ was constructed by connecting a DNA fragment PCR-amplified from pUCORF37-42 using primers ORF39_Dra and Dra_ORF42, with a DNA fragment PCR-amplified from pUCORF43-Km^r^ using primers Dra_ORF43 and ORF44_Dra.

For pUCORF40_DUF35_-Km^r^, two DNA fragments were amplified: one from the Km^r^ gene using primers ORF40_DUF35__Km^r^H and Km^r^T_ORF40_DUF35__2RC, and another from pUCORF37-42 using primers Km^r^T_ORF40_DUF35__2 and ORF40_DUF35__Km^r^HRC. These fragments were assembled using the In-Fusion HD Cloning Kit (TAKARA, Japan).

Plasmids for complementation experiments, such as pMFYChsH2 and pMFYChsH2_DUF35_ (Table S1), were constructed by amplifying DNA fragments containing the target ORF(s) and the Km^r^ gene with suitable primers listed in Table S2. For example, primers MhpRPvuII_ORF40 and ORF40_Km^r^HR were used to amplify the ChsH2 fragment, while primers ORF40_Km^r^H and Km^r^-MFYPvuIIR were used to amplify the Km^r^ fragment. The amplified fragments, along with *Pvu*II-digested pMFYMhpRA, were connected using the In-Fusion HD Cloning Kit.

### UPLC/MS

A 1 mL culture was extracted twice with an equal volume of ethyl acetate under acidic conditions (adjusted to pH 2 with HCl). The ethyl acetate layer was collected, dried, and dissolved in 1 mL of methanol. A 5 µL aliquot of the methanol solution was injected into the UPLC/MS system, Waters Acquity UPLC H-Class-QDa (Waters, Milford, MA, USA).

Chromatographic separation was performed using a reversed-phase column (BEH C18, 2.1 × 50 mm, 1.7 µm; Waters) at a flow rate of 0.6 mL/min. Elution was carried out with 10% solution A (CH_3_CN) and 90% solution B (H_2_O:HCOOH = 100:0.05) for 1.0 min, followed by a linear gradient from 10% solution A and 90% solution B to 80% solution A over 3.0 min, which was maintained for 1 min and back to 10% solution A over 1.5 min, followed by equilibrating for 1 min. Metabolite detection was performed using negative ion electrospray ionization.

## Data Availability

The author affirms that materials and data reasonably requested by other researchers will be made available from a publicly accessible collection or provided in a timely manner at a reasonable cost and in limited quantities to members of the scientific community for noncommercial purposes.

## SUPPLEMENTAL MATERIAL

Supplemental material spectrum.00308-25-s0001.pdf Fig. S1 to S5; Tables S1 and S2.

ASM does not own the copyrights to Supplemental Material that may be linked to, or accessed through, an article. The authors have granted ASM a non-exclusive, world-wide license to publish the Supplemental Material files. Please contact the corresponding author directly for reuse.
