# Supplementary material for: Identification of dehydrogenase, hydratase, and aldolase responsible for the propionyl residue removal in degradation of cholic acid C-17 side chain in Comamonas testosteroni TA441
Source: Microbiol Spectr. 2025 Sep 15;13(10):e00308-25. doi: 10.1128/spectrum.00308-25 (PMC12502768; doi:10.1128/spectrum.00308-25)
Supplement: Supplemental material — Fig. S1 to S5; Tables S1 and S2. [file spectrum.00308-25-s0001.pdf]

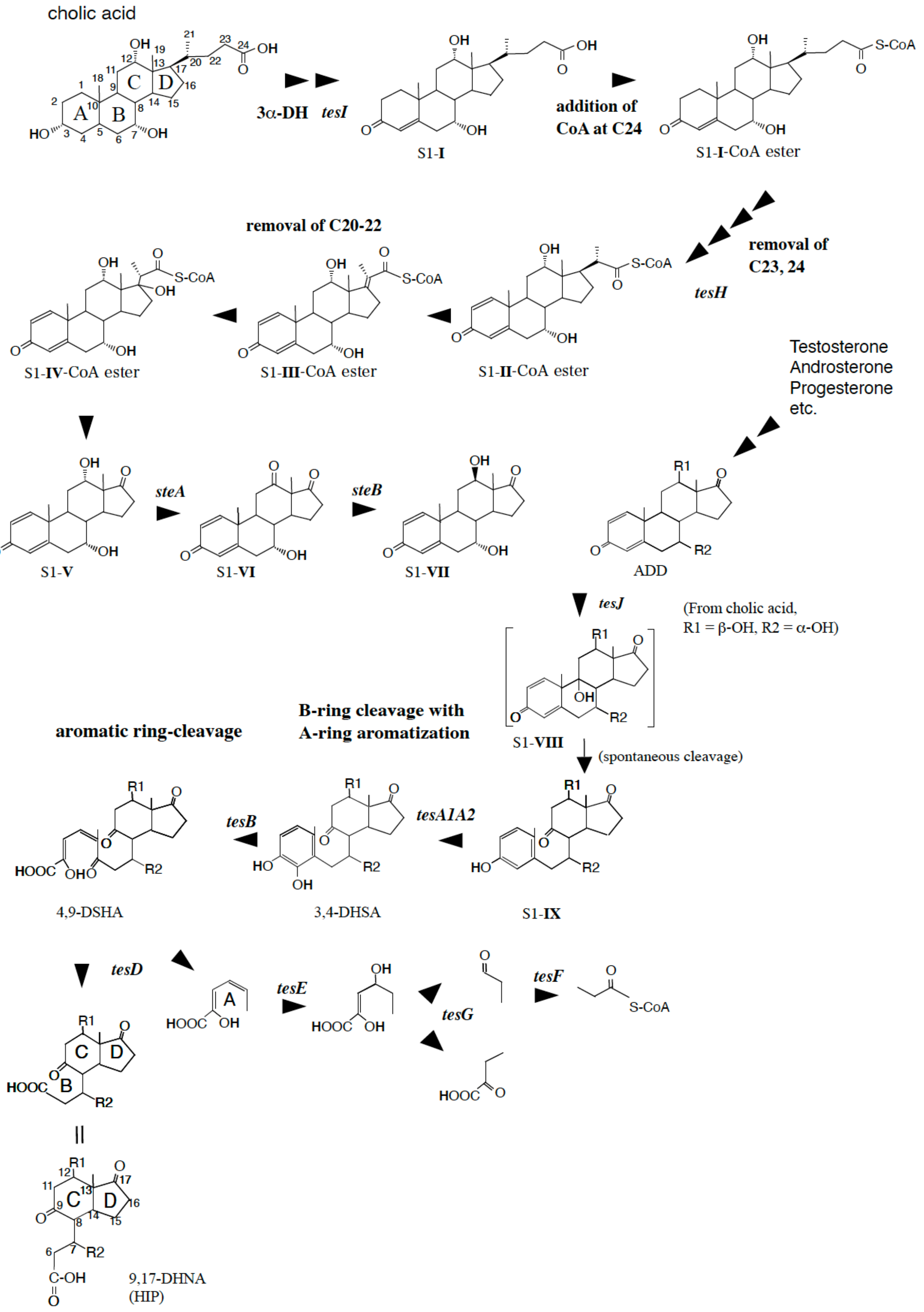
cholic acid
3α-DH tesI
S1-I
addition of CoA at C24
S1-I-CoA ester
removal of C23, 24
tesH
S1-II-CoA ester
S1-III-CoA ester
removal of C20-22
S1-IV-CoA ester
S1-V
steA
S1-VI
steB
S1-VII
Testosterone
Androsterone
Progesterone
etc.
ADD
tesJ
(From cholic acid, R1 = β-OH, R2 = α-OH)
S1-VIII
(spontaneous cleavage)
S1-IX
B-ring cleavage with A-ring aromatization
tesA1A2
3,4-DHSA
tesB
aromatic ring-cleavage
4,9-DSHA
tesD
tesE
tesG
tesF
S-CoA
9,17-DHNA
(HIP)

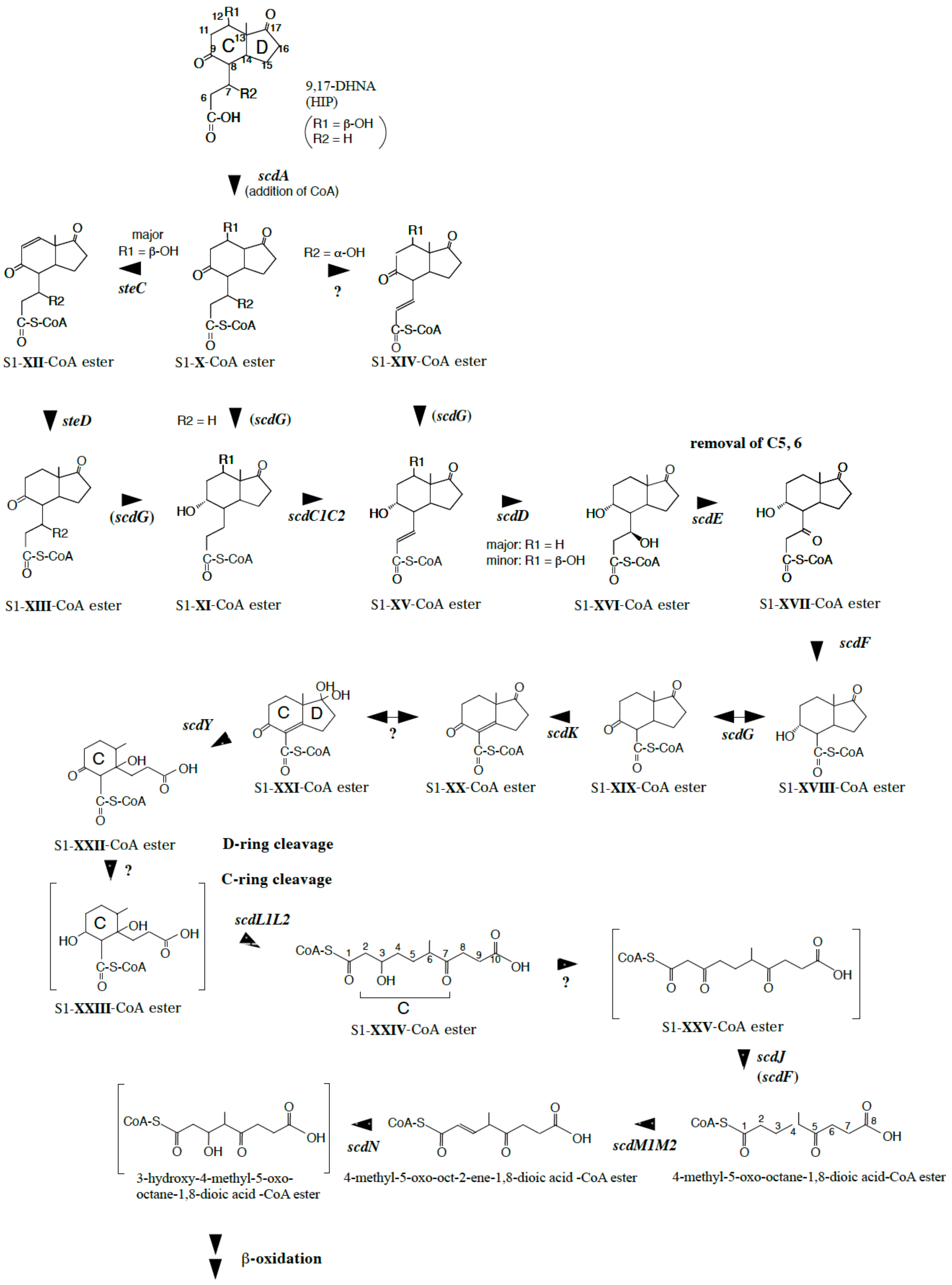

9,17-DHNA
(HIP)
R1 = β-OH
R2 = H
scdA
(addition of CoA)
major
R1 = β-OH
steC
R2 = α-OH
?
S1-XII-CoA ester
S1-X-CoA ester
S1-XIV-CoA ester
steD
R2 = H
(scdG)
(scdG)
removal of C5, 6
(scdG)
scdC1C2
scdD
major: R1 = H
minor: R1 = β-OH
scdE
S1-XIII-CoA ester
S1-XI-CoA ester
S1-XV-CoA ester
S1-XVI-CoA ester
S1-XVII-CoA ester
scdF
scdY
?
scdK
scdG
S1-XXI-CoA ester
S1-XX-CoA ester
S1-XIX-CoA ester
S1-XVIII-CoA ester
S1-XXII-CoA ester
D-ring cleavage
?
C-ring cleavage
scdL1L2
?
S1-XXIII-CoA ester
S1-XXIV-CoA ester
S1-XXV-CoA ester
scdJ
(scdF)
scdN
scdM1M2
3-hydroxy-4-methyl-5-oxo-octane-1,8-dioic acid -CoA ester
4-methyl-5-oxo-oct-2-ene-1,8-dioic acid -CoA ester
4-methyl-5-oxo-octane-1,8-dioic acid-CoA ester
β-oxidation

Fig. S1 Overview of the steroid degradation pathway in *C. testosteroni* TA441. Most compounds were isolated and identified via NMR and mass spectrometry. Compounds with * were confirmed by mass spectrum and conversion experiments, while those in brackets are hypothetical.

Compounds are; 7α,12α-dihydroxy-3-oxo-5β-4-cholen-24-oic acid (S1-**I**), 7α,12α-dihydroxy-3-oxo-5β-4-cholen-24-oic acid-CoA ester (S1-**I**-CoA ester), 7α,12α-dihydroxy-3-oxo-1,4-pregnadine-20-carboxylic acid-CoA ester (S1-**II**-CoA ester), 7α,12α-dihydroxy-3-oxo-1,4,17-pregnatrine-20-carboxylic acid-CoA (S1-**III**-CoA ester), 17-hydroxy-3-oxo-1,4-pregnatriene-20-carboxylic acid-CoA ester (S1-**IV**-CoA ester), 7α,12α-dihydroxy-3,17-dioxo-1,4-androstadien (S1-**V**), 7α-hydroxy-3,12,17-trioxo-1,4-androstadien (S1-**VI**), 7α,12β-dihydroxy-3,17-dioxo-1,4-androstadien (S1-**VII**), 3,17-dioxo-1,4-androstadien (ADD)(R1,R2 = H), 9-hydroxy-3,17-dioxo-1,4-androstadien (S1-**VIII**)(R1, R2=H), 3-HSA:3-hydroxy-9,10-secoandrosta-1,3,5(10)-triene-9,17-dione (S1-**IX**)(R1,R2 = H), 3,4-dihydroxy-9,10-secoandrosta-1,3,5(10)-triene-9,17-dione (3,4-DHSA)(R1,R2 = H), 4,9-DSHA: 4,5-9,10-diseco-3-hydroxy-5,9,17-trioxoandrosta-1(10),2-dien-4-oic acid (R1, R2=H), 9,17-DHNA 9,17-dioxo-1,2,3,4,10,19-hexanorandrostan-5-oic acid (9,17-DHNA) (3aα-*H*-4α [3'-propionic acid]-7aβ-methylhexahydro-1,5-indanedione, HIP), 9,17-dioxo-1,2,3,4,10,19-hexanorandrostan-5-oic acid-CoA ester (R1, R2=H) (S1-**X**-CoA ester), 9α-hydroxy-17-oxo-1,2,3,4,10,19-hexanorandrostan-5-oic acid-CoA ester (R1=H) (S1-**XI**-CoA ester), 9,17-dioxo-1,2,3,4,10,19-hexanorandrost-10(12)-en-5-oic acid-CoA ester (R2=H) (S1-**XII**-CoA ester), 9,17-dioxo-1,2,3,4,10,19-hexanorandrostan-5-oic acid-CoA ester (R2=H) (S1-**XIII**-CoA ester), 9α-hydroxy-17-oxo-1,2,3,4,10,19-hexanorandrost-6-en-5-oic acid-CoA ester (R1=H) (S1-**XIV**-CoA ester), 9α-hydroxy-17-oxo-1,2,3,4,10,19-hexanorandrost-6-en-5-oic acid-CoA ester (R1=H) (S1-**XV**-CoA ester), 7β,9α-dihydroxy-17-oxo-1,2,3,4,10,19-hexanorandrostan-5-oic acid-CoA ester (S1-**XVI**-CoA ester), 9α-hydroxy-7,17-dioxo-1,2,3,4,10,19-hexanorandrostan-5-oic acid-CoA ester (S1-**XVII**-CoA ester), 9α-hydroxy-17-oxo-1,2,3,4,5,6,10,19-octanorandrostan-7-oic acid -CoA ester (S1-**XVIII**-CoA ester), 9,17-dioxo-1,2,3,4,5,6,10,19-octanorandrostan-7-oic acid-CoA ester (S1-**XIX**-CoA ester), 9,17-dioxo-1,2,3,4,5,6,10,19-octanorandrost-8(14)-en-7-oic acid-CoA ester (S1-**XX**-CoA ester), 17-dihydroxy-9-oxo-1,2,3,4,5,6,10,19-octanorandrost-8(14)-en-7-oic acid-CoA ester (S1-**XXI**-CoA ester), 14-hydroxy-9-oxo-1,2,3,4,5,6,10,19-octanor-13,17-secoandrostane-7,17-dioic acid-CoA ester (S1-**XXII**-CoA ester), 9,14-dihydroxy-1,2,3,4,5,6,10,19-octanor-13,17-secoandrostane-7,17-dioic acid (S1-**XXIII**-CoA ester), 3-hydroxy-6-methyl-7-oxo-decane-1,10-dioic acid-CoA ester (S1-**XXIV**-CoA ester), and 6-methyl-3,7-dioxo-decane-1,10-dioic acid-CoA ester (S1-**XXV**-CoA ester)。

Enzymes are; **SteA** (dehydrogenase for 12α-OH to 12-ketone), **SteB** (hydrogenase for 12-ketone to 12β-OH), **TesH** (Δ1-dehydrogenase), **TesI** (Δ4-dehydrogenase), **TesJ** (ADD-hydroxylase at C9), **TesA1A2** (3-HSA-hydroxylase at C4), **TesB** (*meta*-cleavage enzyme for 3.4-DHSA), **TesD** (4.9-DSHA -hydrolase), **TesE** ((2*Z*,4*Z*)-2-hydroxyhexa-2,4-dienoic acid-hydratase), **TesF** (aldolase), **TesG** (acetoaldehyde dehydrogenase), **SteC** (dehydradase for 12β-OH to produce a double at C10(12)), **SteD** (reductase for a double at C10(12) to a single bond), **ScdA** (CoA-transferase for 9,17-dioxo-1,2,3,4,10,19-hexanorandrostan-5-oic acid), **ScdG** (hydrogenase for 9-OH of 9α-hydroxy-17-oxo-1,2,3,4,5,6,10,19-octanorandrostan-7-oic acid-CoA ester), **ScdC1C2** (Δ6-dehydrogenase for 9α-hydroxy-17-oxo-1,2,3,4,10,19-hexanorandrostan-5-oic acid-CoA ester), **ScdD** (9α-hydroxy-17-oxo-1,2,3,4,10,19-hexanorandrost-6-en-5-oic acid -CoA ester Δ6-hydratase), **ScdE** (7β,9α-dihydroxy-17-oxo-1,2,3,4,10,19-hexanorandrostan-5-oic acid-CoA ester dehydrogenase at C7), **ScdF** (9α-hydroxy-7,17-dioxo-1,2,3,4,10,19-hexanorandrostan-5-oic acid-CoA ester thiolase/CoA-transferase), ScdK (Δ8(14)-dehydrogenase for 9,17-dioxo-1,2,3,4,5,6,10,19-octanorandrostan-7-oic acid-CoA ester), **ScdY** (17-dihydroxy-9-oxo-1,2,3,4,5,6,10,19-octanorandrost-8(14)-en-7-oic acid-CoA ester hydratase), **ScdL1L2** (putative CoA-transferase/isomerase necessary for C-ring cleavage of 9,14-dihydroxy-1,2,3,4,5,6,10,19-octanor-13,17-secoandrostane-7,17-dioic acid-CoA ester or maybe C-ring cleavage of 14-hydroxy-9-oxo-1,2,3,4,5,6,10,19-octanor-13,17-secoandrostane-7,17-dioic acid-CoA ester), **ScdJ** (6-methyl-3,7-dioxo-decane-1,10-dioic acid-CoA ester thiolase/CoA-transferase), **ScdM1M2** (4-methyl-5-oxo-octane-1,8-dioic acid-CoA ester dehydrogenase), and **ScdN** (4-methyl-5-oxo-oct-2-ene-1,8-dioic acid -CoA ester hydratase). Genes for the C-, D-, and cleaved B-ring degradation are induced by positive regulator TesR with the compounds possessing steroidal four rings, but are not induced with indane and the derivatives (unpublished data).

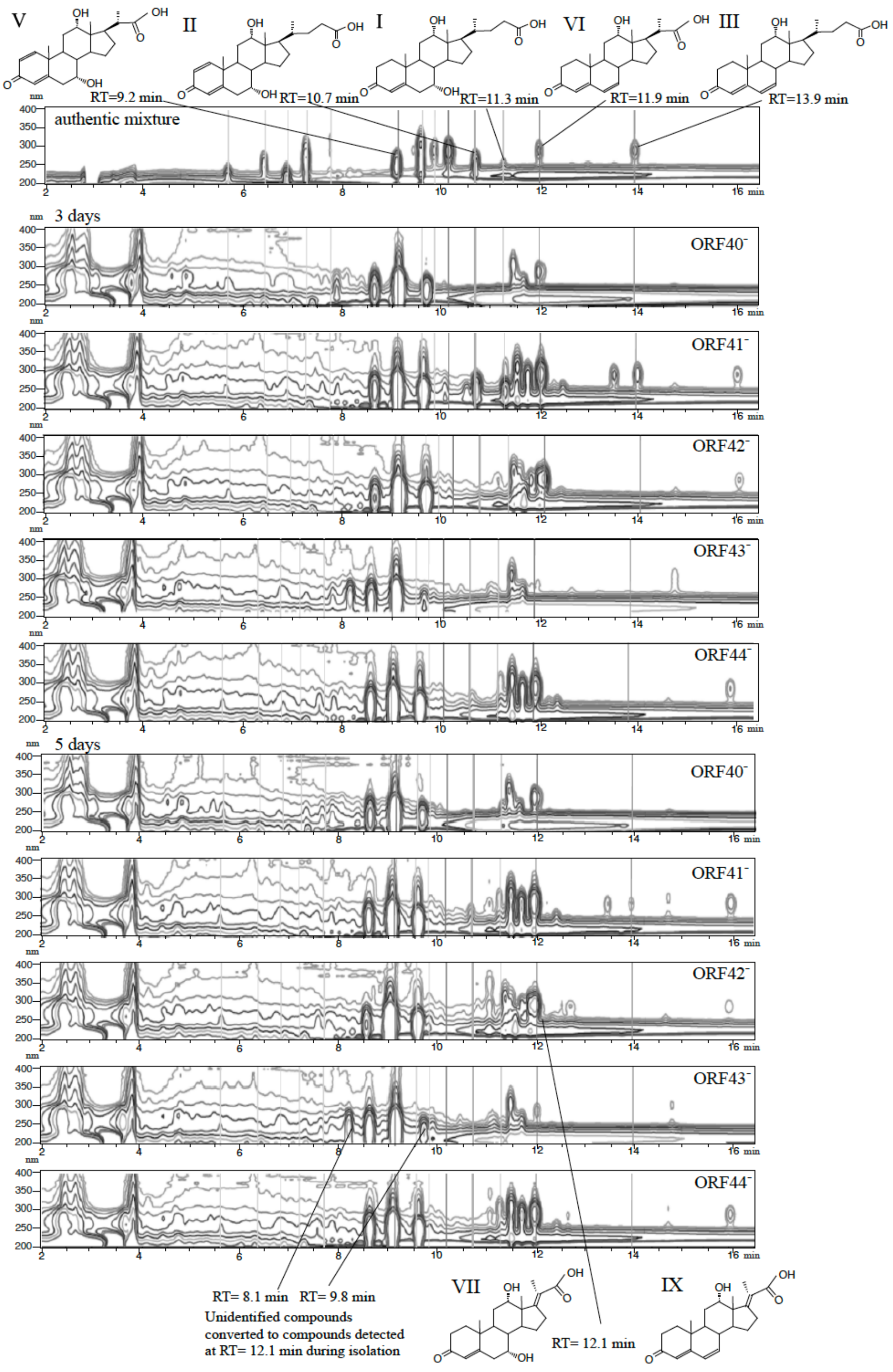

Fig. S2-1 HPLC chromatogram with 3-dimensional (3D) UV detection of intermediate compounds isolated from the culture of the ScdD-disrupted mutant incubated with cholic acid and from mutants individually disrupted in ORF40-44 (referred to as ORF40⁻ to 44⁻), incubated with cholic acid for 3 and 5 days (1). Compounds **I**–**V** were identified based on retention time (RT), UV absorbance, and the predicted functions of the disrupted genes. The peak at RT = 12.1 min contained several unstable compounds, some of which were identified as methyl esters. Compounds **VII** and **IX** were detected in this region and observed only in the ORF40⁻ and ORF42⁻ cultures. Peaks at RT = 8.1 min and 9.8 min were unstable and were converted into compounds eluting at RT = 12.1 min during isolation. The vertical axis indicates wavelength (nm), and the horizontal axis indicates RT (min); UV absorbance is shown in contour format. HPLC was performed using an Alliance 2695 system equipped with an Inertsil ODS-3 column (4.6 × 250 mm, GL Sciences Inc., Tokyo, Japan) and UV and 996 photodiode array detectors (Nihon Waters, Tokyo, Japan). Elution was carried out with a linear gradient from 20% solution A (CH_3CN:CH_3OH:TFA = 95:5:0.05; TFA = trifluoroacetic acid) and 80% solution B (H_2O:CH_3OH:TFA = 95:5:0.05) to 65% solution A and 35% solution B over 10 minutes, held for 3 minutes, and returned to 20% solution A. The flow rate was 1.0 mL/min at 40°C.

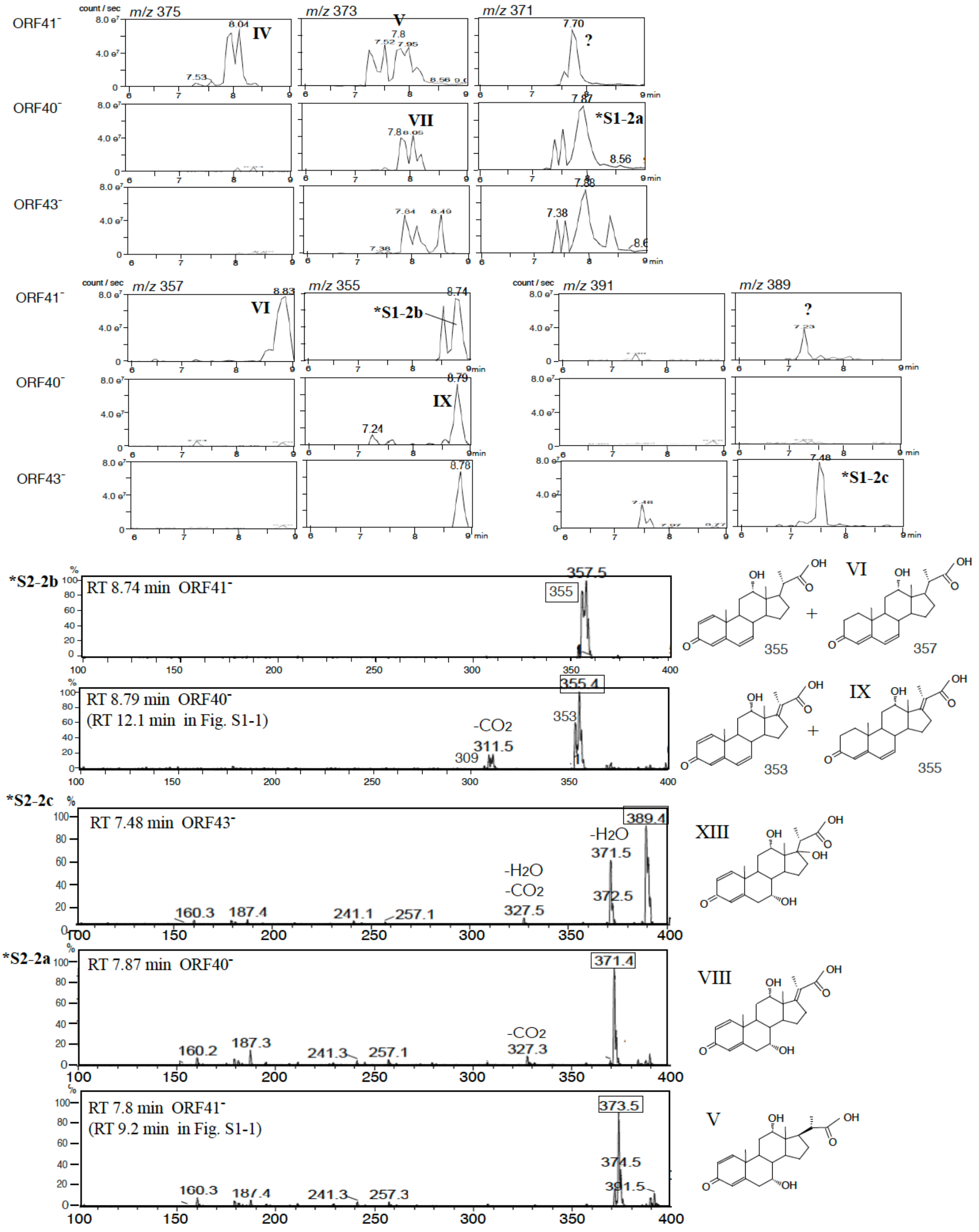

Fig. S2-2 HPLC chromatograms and mass spectra of compounds detected in cultures of ORF40$^-$, ORF41$^-$, andORF43$^-$ mutants incubated with cholic acid (data for ORF44$^-$ and ORF42$^-$ are not shown, as they were essentially identical to ORF41$^-$ and ORF40$^-$, respectively). ***S2-2a***: Fragmentation patterns were nearly identical, except for fragments at *m/z* 327 and *m/z* 371. The fragment at *m/z* 327 corresponds to a 3-enoyl carboxylic acid, identifying the compound as **VIII**. ***S2-2b***: This compound, detected only in ORF41$^-$ and ORF44$^-$ cultures, showed only a molecular ion peak and eluted near **VI**, suggesting a structure similar to **VI** but with a double bond at C1. Similarly, a compound with a structure analogous to **IX** but with a double bond at C1 was detected. ***S2-2c***: Accumulated only in the ORF43$^-$ mutant culture, this compound showed a fragmentation pattern nearly identical to **VIII**, except for the parent ion. A large peak at *m/z* 371 indicates the presence of an unstable hydroxyl group, suggesting that the compound is structurally similar to **VIII** but with a hydroxyl group at C17 (designated **XIII**). The vertical axis indicates wavelength (nm), and the horizontal axis indicates RT (min). For LC/MS analysis, 2 μL of each sample (prepared as for HPLC/MS) was injected into an Agilent 1100 HPLC system (Agilent, CA) coupled to a 4000 QTRAP MS/MS system (AB SCIEX, Framingham, MA, USA) operating in negative ion mode, with an L-column2 ODS (1.5 × 150 mm, 5 μm, GL Sciences, Tokyo, Japan). Elution was performed with 90% solution A (H_2O:HCOOH = 100:0.1) and 10% acetonitrile for 1 minute, followed by a linear gradient to 20% solution A and 80% acetonitrile over 7 minutes, held for 2 minutes. The flow rate was 0.2 mL/min at 40°C.

CA

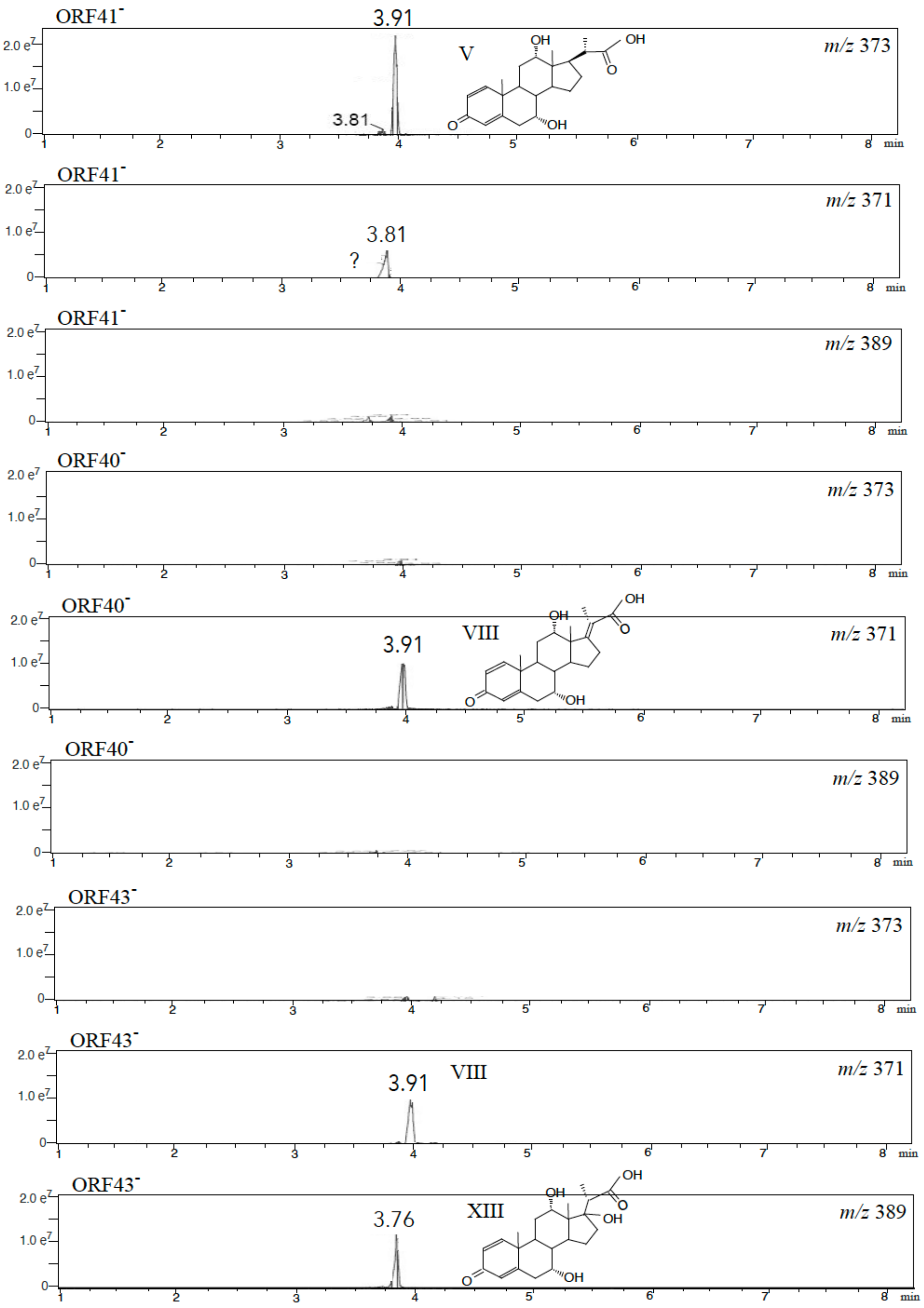

Fig. S2-3CA HPLC chromatograms (RT = 1–8 min) of cultures of ORF40⁻, ORF41⁻, and ORF43⁻ mutants incubated with cholic acid (data for ORF42⁻ and ORF44⁻ are not shown, as they were essentially identical to ORF40⁻ and ORF41⁻, respectively), analyzed using a Waters Acquity UPLC H-Class QDa system. A single major peak of *m/z* 373, 371, or 389 was detected in the respective cultures, corresponding to **V**, **VIII**, and **XIII**. Chromatographic separation was performed using a BEH C18 reversed-phase column (2.1 × 50 mm, 1.7 μm, Waters) at a flow rate of 0.6 mL/min. Elution was carried out with 10% solution A (CH_3CN) and 90% solution B (H_2O:HCOOH = 100:0.05) for 1 minute, followed by a linear gradient to 80% solution A over 3 minutes, held for 1 minute, and returned to 10% solution A over 1.5 minutes, followed by 1 minute of re-equilibration. Metabolites were detected using negative ion electrospray ionization.

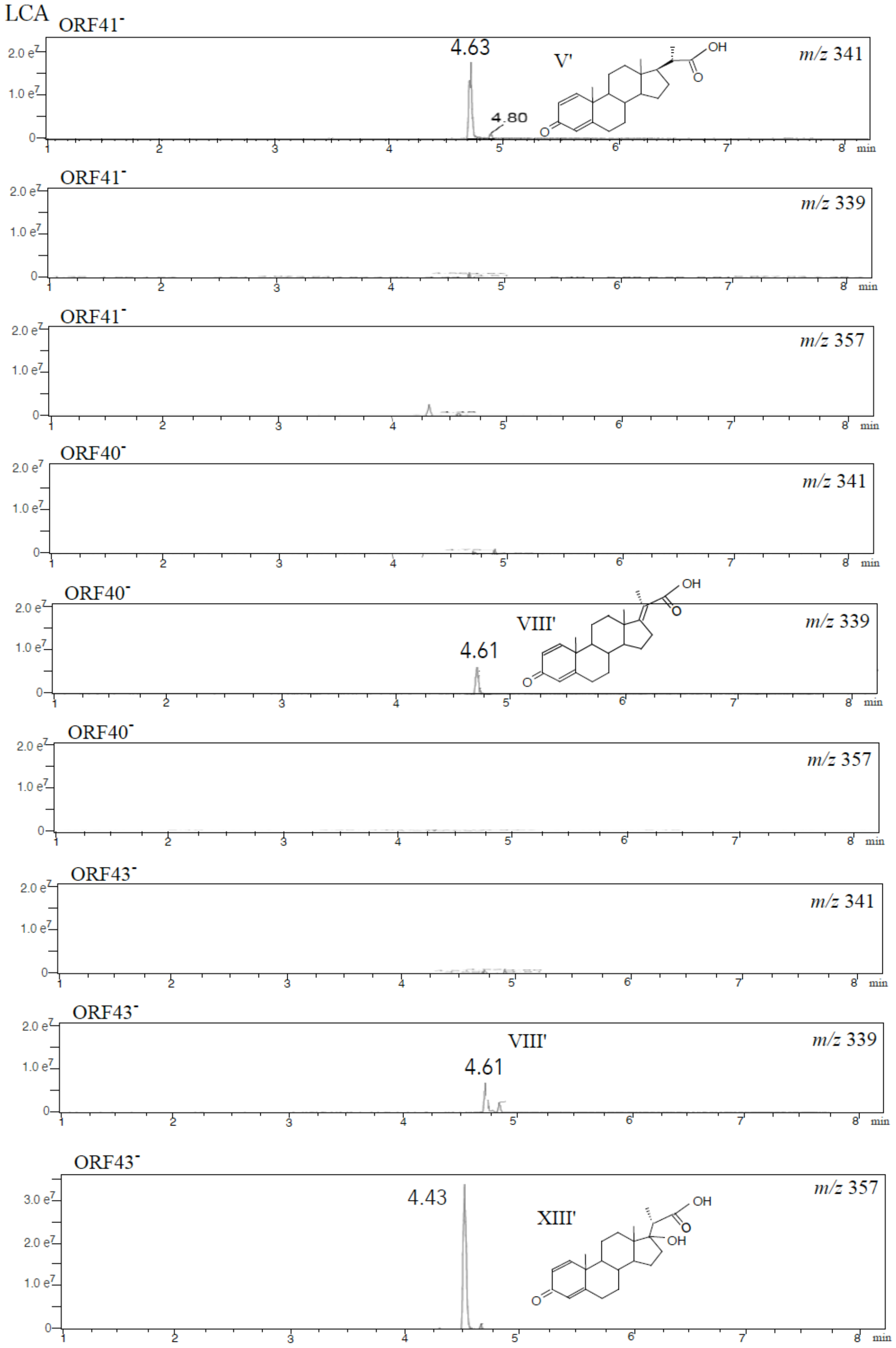

Fig. S2-3LCA HPLC chromatograms (RT = 1–8 min) of cultures of ORF40^{-}, ORF42^{-}, and ORF43^{-} mutants incubated with lithocholic acid (LCA) (data for ORF41^{-} and ORF44^{-} are not shown). The results were similar to those obtained with cholic acid: a single major peak at *m/z* 341, 339, or 357 was observed in ORF40^{-}, ORF42^{-}, and ORF43^{-} cultures, respectively, corresponding to **V′**, **VIII′**, and **XIII′**. Chromatographic conditions and detection methods were the same as those used in Fig. S2-3CA.

ORF41$^-$ RT=4.62min

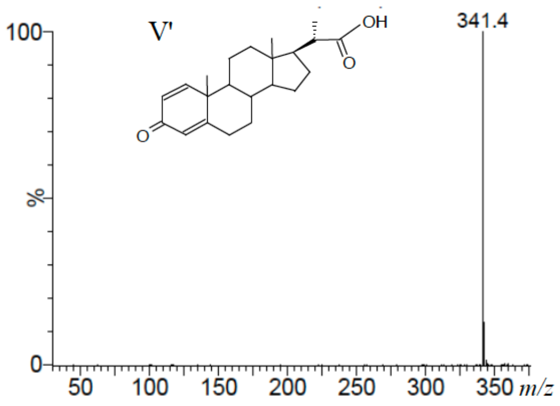

ORF42$^-$ RT=4.62min

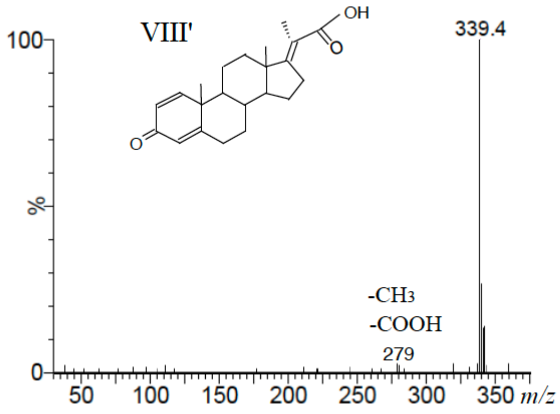

ORF43$^-$ RT=4.43min

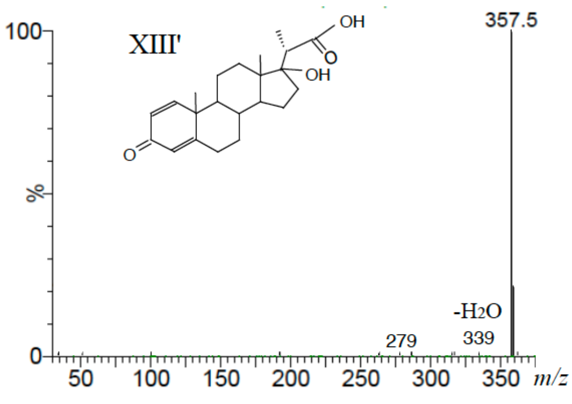

Fig. S2-4 Mass spectra of the major peaks at *m/z* 341, 339, and 357 detected in Fig. S2-3LCA. Although primarily showing molecular ion peaks (due to constraints on instrument conditions), the observed minor fragments were consistent with the structures of **V′**, **VIII′**, and **XIII′**.

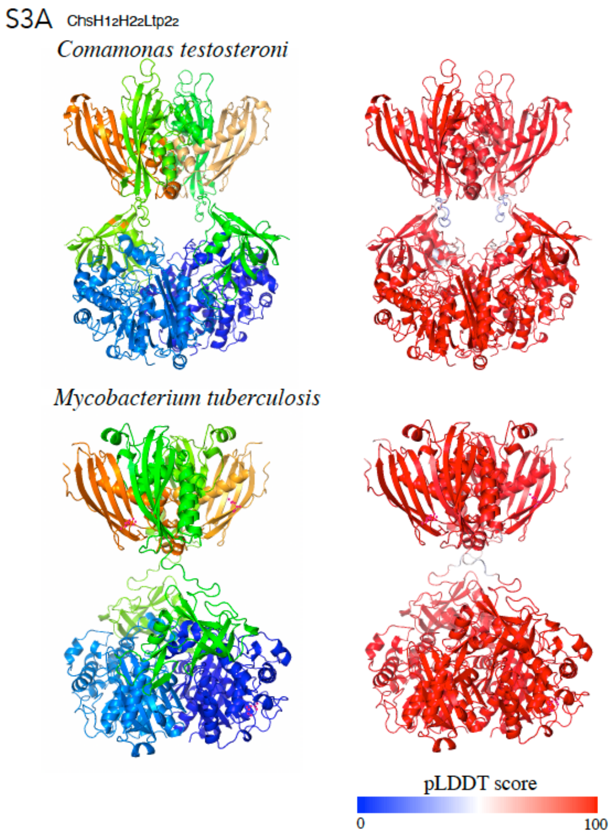

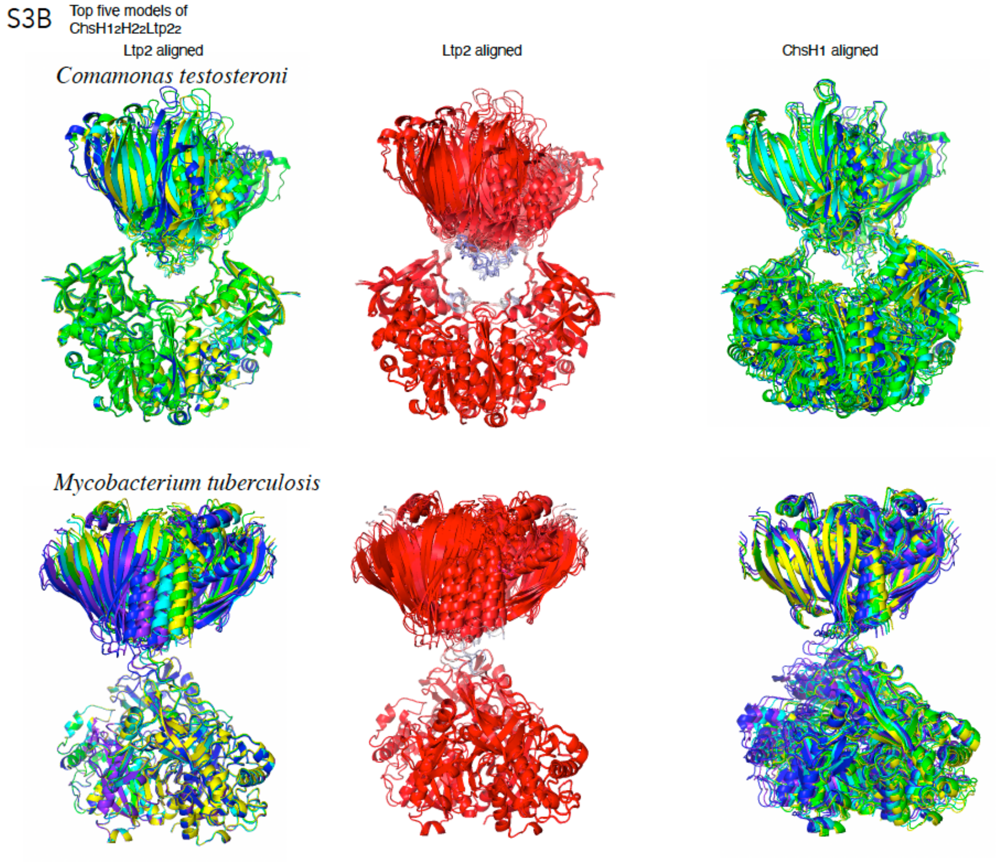

Fig. S3 (A) AlphaFold-predicted multimer models of the (ChsH1–ChsH2)$_2$Ltp2$_2$ complex from *C. testosteroni* TA441 and *M. tuberculosis*, with per-residue pLDDT confidence scores indicated by a blue–white–red color gradient (0–100%).
(B) Structural alignments of the top five AlphaFold multimer models of the (ChsH1–ChsH2)$_2$Ltp2$_2$ complex in *C. testosteroni* TA441 and *M. tuberculosis*. The Ltp2-aligned model, the pLDDT score distribution, and the ChsH1-aligned model are shown.

Comamonas testosteroni

ChsE1$_2$E2$_2$H2$_2$H1$_2$Ltp2$_2$

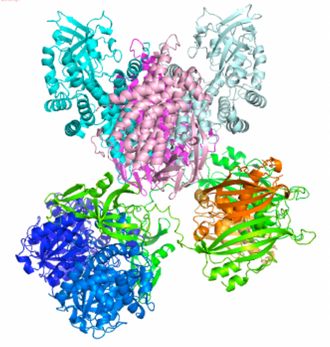

	subunits		dehydrogenase	hydratase	aldorase
S4A ChsH2$_{MaoC}^-$ + pMFYMhpRA (Fig. 4C) 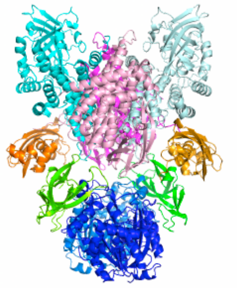	(ChsE1-ChsE2)$_2$ (ChsH1-ChsH2$_{DUF35}$)$_2$	+Tc	-	*	*
		-Tc	+	-	*
S4B ChsH2$_{DUF35}^-$ + pMFYMhpRA (Fig. 4E) 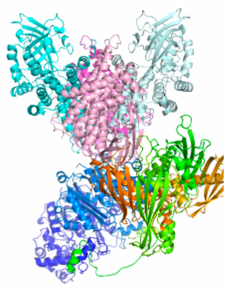 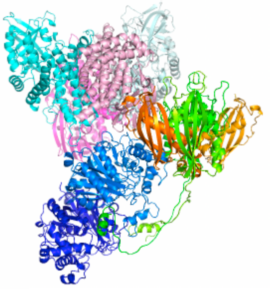	(ChsE1-ChsE2)$_2$ (ChsH1-ChsH2$_{MaoC}$)$_2$	+Tc	+	+	-
S4C ChsH1$^-$ + pMFYMhpRA (Fig. 4D) 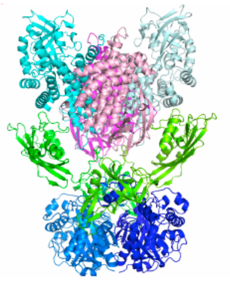 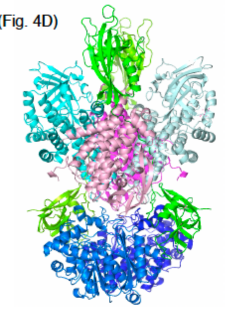	(ChsE1-ChsE2)$_2$ ChsH2$_2$ Ltp2$_2$	+Tc	-	*	*
		-Tc	+	-	*

		subunits		dehydrogenase	hydratase	aldorase
S4D	ChsE1$^-$H1$^-$H2$^-$Ltp2$^-$ + pMFYMhpChsE1H2(Fig. 5C)<br>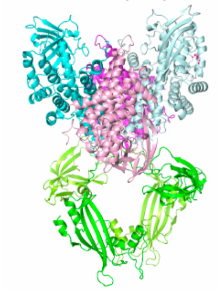	(ChsE1-ChsE2)$_2$<br>ChsH2$_2$	+Tc	±	*	*
S4E	ChsE1$^-$H1$^-$H2$^-$Ltp2$^-$ + pMFYMhpChsE1H1 (Fig. 5D)<br>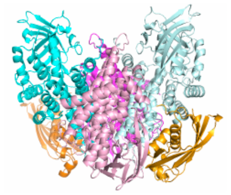	(ChsE1-ChsE2)$_2$<br>ChsH1$_2$	+Tc	-	*	*
S4F	ChsE1$^-$H1$^-$H2$^-$Ltp2$^-$ + pMFYMhpChsE1H1H2 (Fig. 5G)<br>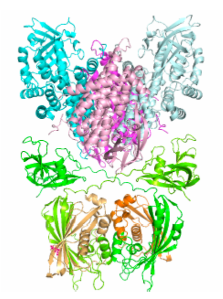 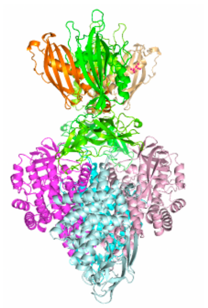	(ChsE1E2)$_2$<br>(ChsH1H2)$_2$	+Tc	+	+	-

Fig. S4 Additional AlphaFold-predicted multimer models of protein complexes expressed in the mutants used in Figs. 4 and 5 are shown with the enzyme activity detected in the experiment. For each case, a representative model in five top models presented by AlphaFold is shown, and if an alternative model differed substantially, it is also included on the right. The color scheme used in the structural models is as follows: cyan/light cyan for ChsE1, magenta/light magenta for ChsE2, orange/light orange for ChsH1, green/light green for ChsH2 (or ChsH2$_{MaoC}$ when subdivided), moss green/light moss green for ChsH2$_{DUF}$, and blue/light blue for Ltp2. S4A: the model of the expected complex in ChsH2$_{MaoC}^-$ChsE1$^-$ChsH1$^-$Ltp2$^-$ carrying pMFYMhpRA (mutant in Fig. 4C); S4B: the model of the expected complex in ChsH2$_{DUF}^-$ChsE1$^-$ChsH1$^-$Ltp2$^-$ carrying pMFYMhpRA (mutant in Fig. 4E), along with an alternative model that differed significantly; S4C: the model of the expected complex in ChsH1$^-$ChsE1$^-$ChsH2$^-$Ltp2$^-$ carrying pMFYMhpRA (mutant in Fig. 4D); S4D: the model of the expected complex in ChsE1$^-$ChsH1$^-$ChsH2$^-$Ltp2$^-$ carrying pMFYMhpChsE1H2 (mutant in Fig. 5C); S4E: the model of the expected complex in ChsE1$^-$ChsH1$^-$ChsH2$^-$Ltp2$^-$ carrying pMFYMhpChsE1H1 (mutant in Fig. 5D); S4F: the model of the expected complex in ChsE1$^-$ChsH1$^-$ChsH2$^-$Ltp2$^-$ carrying pMFYMhpChsE1H1H2 (mutant in Fig. 5G), with an alternative model included.

Expected Position Error

Comamonas testosteroni

S5A1 (for Fig. 6A1)

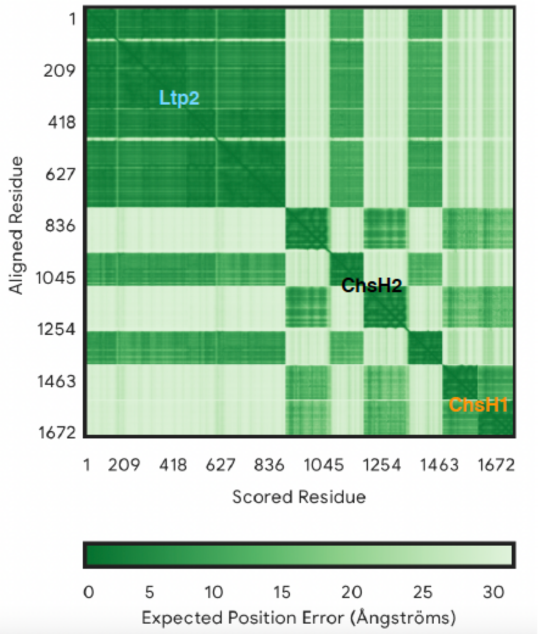

Mycobacterium tuberculosis

S5B1 (for Fig. 6B1)

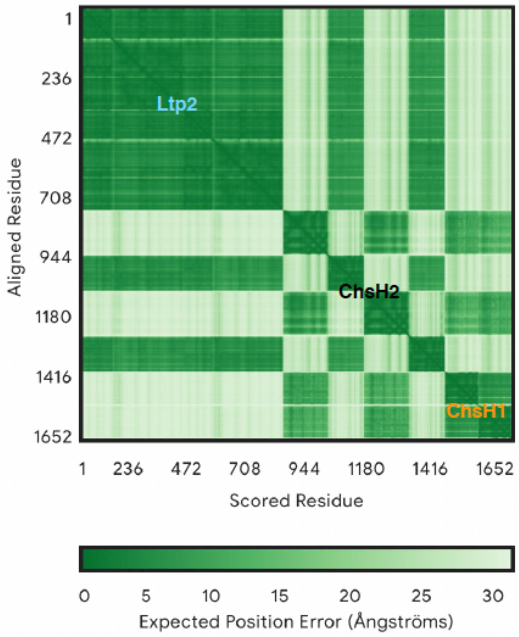

S5A2 (for Fig. 6A2)

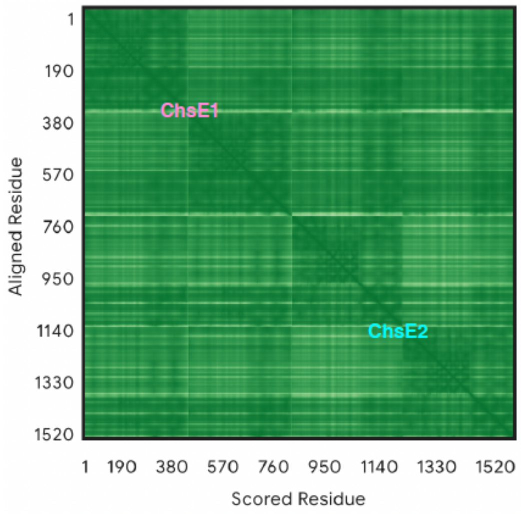

S5B2 (for Fig. 6B2)

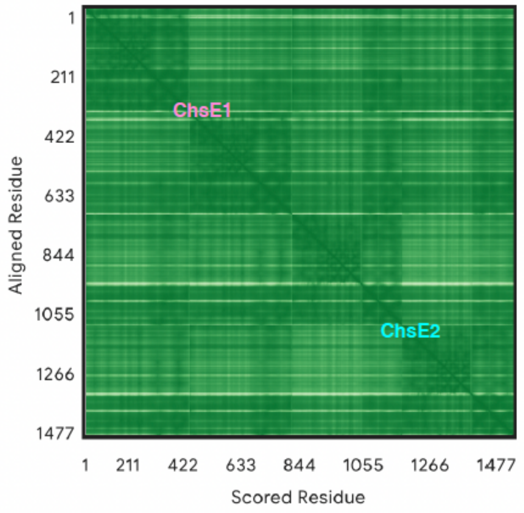

S5A3 (for Fig. 6A3)

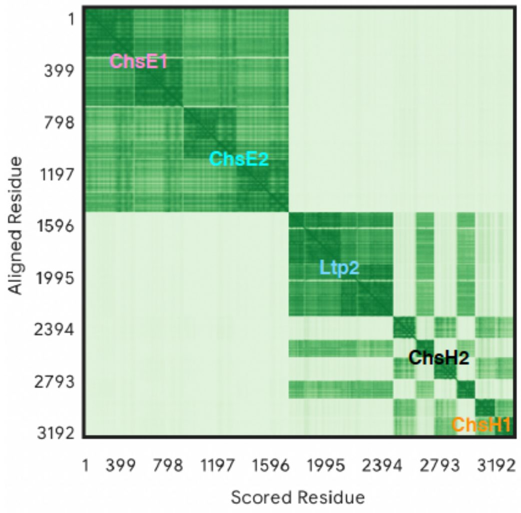

S5B3 (for Fig. 6B3)

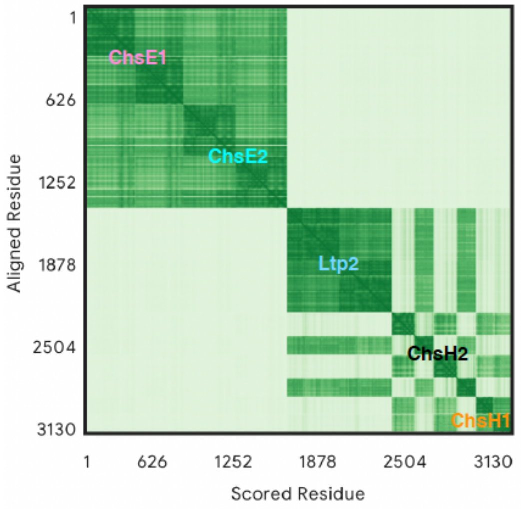

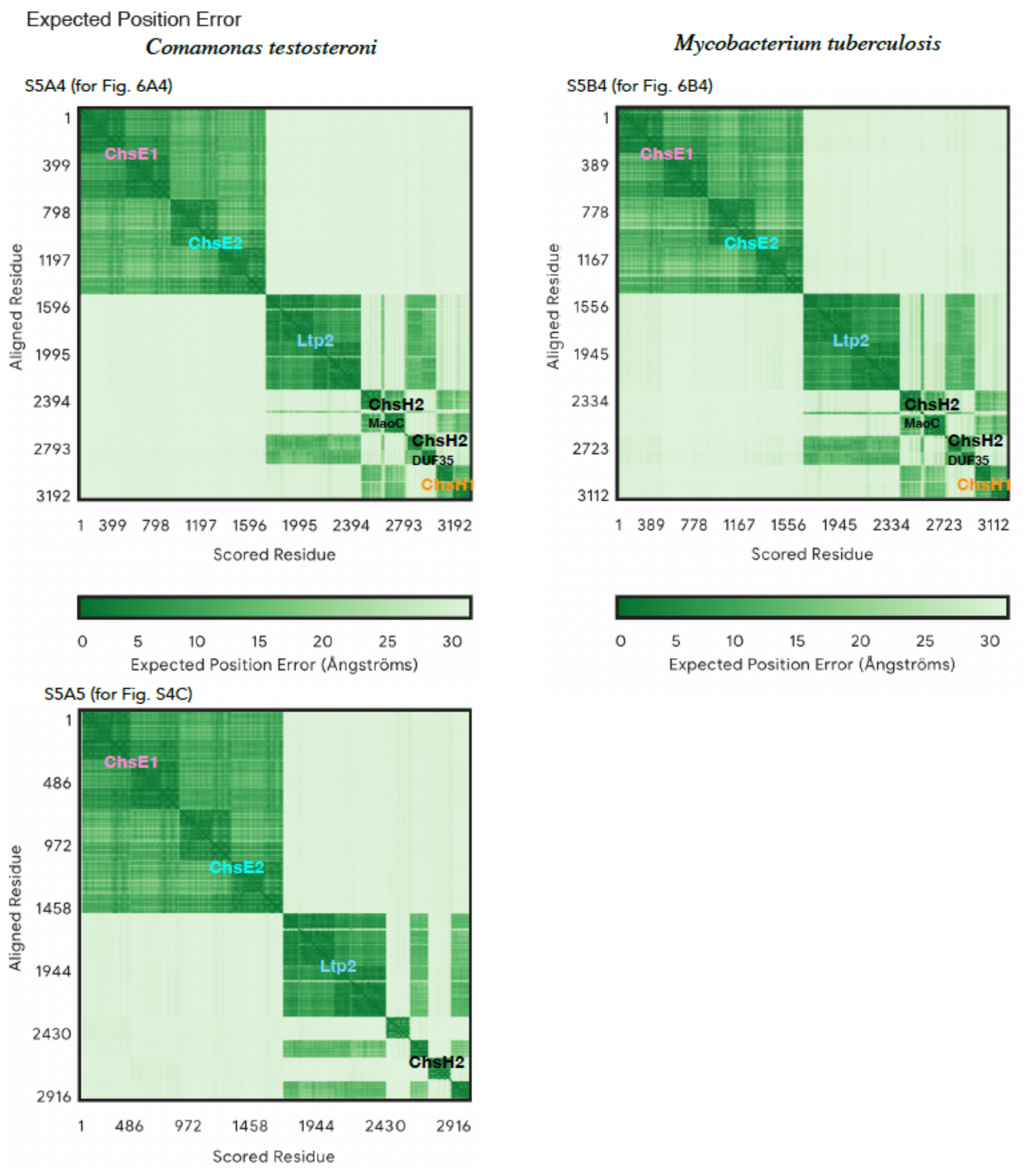

Fig. S5 Expected position error maps of the AlphaFold multimer models shown in Fig. 6 and Fig. S4C. Darker green indicates stronger predicted interactions between amino acid

Table S1 plasmids

plasmids	Characteristics	Source or reference
pUC19	Ap^r, *lac*Z	(2)
pMFY42	Tc^r, Km^r, RSF1010-based broad host range plasmid	(3)
pMFYMhpRA	pMFY42 derivative carrying *mhpR* and the promoter of *mhp* genes	(4)
pMFYMhpChsH2	pMFYMhpRA derivative carrying *chsH2*	this work
pMFYMhpChsE1	pMFYMhpRA derivative carrying *chsE1*	this work
pMFYMhpChsH1	pMFYMhpRA derivative carrying *chsH1*	this work
pMFYMhpLtp2	pMFYMhpRA derivative carrying *ltp2*	this work
pMFYMhpChsE2	pMFYMhpRA derivative carrying *chsE2*	this work
pMFYMhpChsE1H2	pMFYMhpRA derivative carrying *chsE1* and *chsH2*	this work
pMFYMhpChsE1H1	pMFYMhpRA derivative carrying *chsE1* and *chsH1*	this work
pMFYMhpChsE1H1H2	pMFYMhpRA derivative carrying *chsE1H1H2*	this work
pMFYMhpChsE1H1H2Ltp2	pMFYMhpRA derivative carrying *chsE1H1H2ltp2*	this work
pMFYMhpChsH2$_{MaoC}$	pMFYMhpRA derivative carrying MaoC domain of *chsH2*	this work
pMFYMhpChsH2$_{DUF35}$	pMFYMhpRA derivative carrying DUF35 domain of *chsH2*	this work
pHSG10-2-8-43	pHSG397 derivative carrying *Eco*RI fragment containing ORF35 to 39	this work
pHSG10-2-8-28	pHSG397 derivative carrying *Eco*RI fragment containing ORF41 to 45	this work
pUC37-42	pUC derivative carrying DNA fragment containing ORF37 to 42	this work
pUCORF40-Km^r	pUC19 derivative carrying DNA fragment containing ORF40$_{MaoC}$: :Km^r	this work
pUCORF41-Km^r	pUC19 derivative carrying DNA fragment containing ORF41: :Km^r	this work
pUCORF42-Km^r	pUC19 derivative carrying DNA fragment containing ORF42: :Km^r	this work
pUCORF43-Km^r	pUC19 derivative carrying DNA fragment containing ORF43: :Km^r	this work
pUCORF44-Km^r	pUC19 derivative carrying DNA fragment containing ORF44: :Km^r (*Eco*RV site)	this work
pUCORF40$_{DUF35}$-Km^r	pUC19 derivative carrying DNA fragment containing ORF40$_{DUF35}$: :Km^r	this work
pUCORF40-43-Km^r	pUC19 derivative carrying DNA fragment containing ORF40-43: :Km^r	this work

Km^r : Km-resistance
pSuperCosI* (Stratagene, CA)

(2) Vieira, J., and Messing, J. 1987. Methods Enzymol. **153:** 3-11.
(3) Nagata Y. et al. 1993. J Bacteriol **175**:6403-6410
(4) Horinouchi M, et al. 2023. Appl Environ Microbiol **89**:e0105023.

Table S2 primers

primers	Sequences	Source or reference
Dra_ORF40	TTTAAATGGCAGAGGTCCGCGCGTA	this work
Dra_ORF41	TTTAAATGGACTTTCAACTCAGTG	this work
Dra_ORF42	TTTAAATGGCAAACCAATCTATTCG	this work
Dra_ORF43	TTTAA ATGAATATTTCCGGTCGCGC	this work
Dra_ORF44	TTTAAATGTTCATTGACCTGACTTC	this work
ORF39_Dra	TTTAAAGTGTTTTTGTTGCACGGATT	this work
ORF40_Dra	TTTAAAGCCCACGGGACGGAACTGGG	this work
ORF41_Dra	TTTAAAGCGGTCTTCGGGCAGGTCGT	this work
ORF42_Dra	TTTAAAGGCCAGGGCCACTTGCACGG	this work
ORF43_Dra	TTTAAAGGCCTCATCGCCCAGGATCA	this work
ORF44_Dra	TTTAAAGCGCTGAGGGCGCGGCATGC	this work
ORF40_Km^r	GCCTGGGACAAG**GTTAAC***AGCCCCATGATT	this work
ORF40_Km^{r}RC	AATCATGGGGCT**GTTAAC***CTTGTCCCAGGC	this work
ORF41_Km^r	CAGTGGCTCGCA**GTTAAC***GACACGCTCGGCTGG	this work
ORF41_Km^{r}RC	CCAGCCGAGCGTGTC**GTTAAC***TGCGAGCCACTG	this work
ORF42_Km^r	CACGCGAGCTGG**GTTAAC***CCCATATCTTCA	this work
ORF42_Km^{r}RC	TGAAGATATGGG**GTTAAC***CCAGCTCGCGTG	this work
ORF43_Km^r	CGTGGCAGCTCC**GTTAAC***CAGGTCAAGGA	this work
ORF43_Km^{r}RC	TCCTTGACCTG**GTTAAC***GGAGCTGCCACG	this work
ORF40DUF35_Km^{r}H	GCGTGCGTCTGATTGCACGCTGCCGCAAGC	this work
ORF40DUF35_Km^{r}HRC	GCTTGCGGCAGCGTGCAATCAGACGCACGC	this work
Km^{r}T_ORF40DUF35_2	GACGAGTTCTTCTGAGCAAGAGGGCGTGCG	this work
Km^{r}T_ORF40DUF35_2RC	CGCACGCCCTCTTGCTCAGAAGAACTCGTC	this work
MhpRPvuII_ORF40	GAGAATCTGGCCCAGATGGCAGAGGTCCGC	this work
MhpRPvuII_ORF41	GAGAATCTGGCCCAGATGGACTTTCAACTCA	this work
MhpRPvuII_ORF42	GAGAATCTGGCCCAGATGGCAAACCAATCT	this work
MhpRPvuII_ORF43	GAGAATCTGGCCCAGATGAATATTTCCGGT	this work
MhpRPvuII_ORF44	GAGAATCTGGCCCAGATGTTCATTGACCTG	this work
MhpRPvuII_ORF40MaoC	GAGAATCTGGCCCAGGGAGTACTTCTCGAT	this work
ORF40_Km^{r}HR	GCGCTGAGGGCGCGGGCCCACGGGACGGAA	this work
ORF41_Km^{r}H	CAGTGGCTCGCAGTTAACGACACGCTCGGCTGG	this work
ORF41_Km^{r}HRC	CCAGCCGAGCGTGTCGTTAACTGCGAGCCACTG	this work
ORF42_Km^{r}H	CCGCACGCGAGCTGGGTTAACCCCATATCTTCA	this work
ORF42_Km^{r}HRC	TGAAGATATGGGGTTAACCCAGCTCGCGTGCGG	this work
ORF42_2_Km^{r}H	GCGCTGAGGGCGCGGGGCCAGGGCCACTTG	this work
ORF43_Km^{r}HR	GCGCTGAGGGCGCGGGGCCTCATCGCCCAG	this work
ORF44_Km^r	GATTCAGACCGTGGGCACGCTGCCGCAAGC	this work
ORF44_Km^{r}RC	GCTTGCGGCAGCGTGCCCACGGTCTGAATC	this work
ORF44D_Km^r	GAAGACATCTCACACCTGCAGGAATTCGAT	this work
ORF44D_Km^{r}RC	ATCGAATTCCTGCAGGTGTGAGATGTCTTC	this work
ORF41_44	CTGCCCGAAGACCGCATGTTCATTGACCTG	this work
ORF41_44RC	CAGGTCAATGAACATGCGGTCTTCGGGCAG	this work
ORF42_44	CAAGTGGCCCTGGCCATGTTCATTGACCTG	this work
ORF42_44RC	CAGGTCAATGAACATGGCCAGGGCCACTTG	this work
ORF42-43	CAAGTGGCCCTGGCCATGAATATTTCCGGTC	this work
ORF42-43RC	GACCGGAAATATTCATGGCCAGGGCCACTTG	this work
ORF43_44	GCAAGGGAGACATTCATGTTCATTGACCTG	this work
ORF43_44RC	CAGGTCAATGAACATGAATGTCTCCCTTGC	this work
ORF40DUF35-ORF41	GCGTGCGTCTGATTGATGGACTTTCAACTC	this work
ORF40DUF35-ORF41RC	GAGTTGAAAGTCCATCAATCAGACGCACGC	this work
ORF40DUF35in-Km^{r}H	GCGTGCGTCTGATTGCACGCTGCCGCAAGC	this work
ORF40DUF35in-Km^{r}HR	GCTTGCGGCAGCGTGCAATCAGACGCACGC	this work
Km^r-MFYPvuII R	GGGGTGGGCGAAGAACTGGCAATTCCGGTT	this work

Km^r : Km-resistance
***GTTAAC**: *Hpa*I site

REFERENCES

1. Horinouchi M, Hayashi T, Koshino H, Malon M, Hirota H, Kudo T. 2014. Identification of 9alpha-hydroxy-17-oxo-1,2,3,4,10,19-hexanorandrost-6-en-5-oic acid and beta-oxidation products of the C-17 side chain in cholic acid degradation by *Comamonas testosteroni* TA441. *J Steroid Biochem Mol Biol* 143:306-322.
2. Vieira J, Messing J. 1987. Production of single-stranded plasmid DNA. *Methods Enzymol* 153:3-11.
3. Nagata Y, Nariya T, Ohtomo R, Fukuda M, Yano K, Takagi M. 1993. Cloning and sequencing of a dehalogenase gene encoding an enzyme with hydrolase activity involved in the degradation of g-hexachlorocyclohexane (g-HCH) in *Pseudomonas paucimobilis*. *J Bacteriol* 175:6403-6410.
4. Horinouchi M, Hayashi T. 2023. Identification of "missing links" in C- and D-ring cleavage of steroids by *Comamonas testosteroni* TA441. *Appl Environ Microbiol* 89:e0105023.
